# Synthetic trap‐peptides identify a TOM complex phosphatase – PP2A dephosphorylates Tom6

**DOI:** 10.1111/febs.70246

**Published:** 2025-09-02

**Authors:** Laura Scheinost, Christina Ludwig, Nico Höfflin, Asli Aras Taskin, Adinarayana Marada, F.‐Nora Vögtle, Chris Meisinger, Maja Köhn

**Affiliations:** ^1^ Faculty of Biology, Institute of Biology III University of Freiburg Germany; ^2^ Signalling Research Centres BIOSS and CIBSS University of Freiburg Germany; ^3^ Spemann Graduate School of Biology and Medicine (SGBM) University of Freiburg Germany; ^4^ Department of Molecular Cell Biology, Institute for Cell Biology University of Bonn Germany; ^5^ Chair of Proteomics and Bioanalytics, TUM School of Life Sciences Technical University of Munich (TUM) Freising Germany; ^6^ Bavarian Center for Biomolecular Mass Spectrometry (BayBioMS), TUM School of Life Sciences Technical University of Munich (TUM) Freising Germany; ^7^ Institute of Biochemistry and Molecular Biology, ZBMZ, Faculty of Medicine University of Freiburg Germany; ^8^ Center for Molecular Biology of Heidelberg (ZMBH), DKFZ‐ZMBH Alliance University of Heidelberg Germany; ^9^ Network Aging Research Heidelberg University Germany; ^10^ CIBSS ‐ Centre for Integrative Biological Signalling Studies University of Freiburg Germany

**Keywords:** PP2A, PP4, TOM complex, Tom6 Phosphatase substrates

## Abstract

The identification of phosphatases that dephosphorylate specific sites in proteins remains a major challenge, particularly for the major class of serine/threonine‐specific phosphatases, which function as holoenzymes. Here, we report the development of synthetic trap‐peptides to identify phosphatases that bind to Tom6, a subunit of the mitochondrial translocase of the outer membrane (TOM) complex. The TOM complex is regulated by reversible phosphorylation, and although responsible kinases have been identified, the corresponding phosphatases so far remain unknown. Here, the trap‐peptides enriched phosphoserine/threonine‐specific protein phosphatases 2A (PP2A) and 4 (PP4) as full holoenzymes from yeast cytosolic fractions. We observed that their interaction with Tom6 was mediated through their regulatory subunits Cdc55^reg^ and Psy2^reg^, respectively, and that PP2A was able to dephosphorylate Ser16 of Tom6 *in vitro*. In summary, synthetic trap‐peptides facilitate the identification of complete holoenzymes that bind to the target sequence and reveal PP2A as the first TOM phosphatase.

## Graphical abstract

Synthetic trap‐peptides facilitate the identification of complete holoenzymes that bind to the target sequence remarkably close to the phosphorylation site. PP4 bound to, but did not dephosphorylate, the peptide substrate taken from yeast Tom6 pSer16, showing that proximity alone does not induce phosphatase activity. PP2A/Cdc55 is the first known phosphatase to dephosphorylate a protein of the TOM complex, providing important insights into the regulation of the mitochondrial protein biogenesis process.

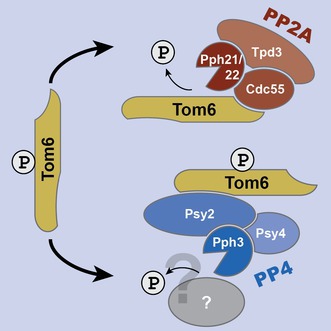

**Abbreviations**
Ac: acetyl
Ac_2_O: acetic anhydride
ACN: acetonitrile
AGC: automatic gain control
Am: amide
AP: alkaline phosphatase
BrTMS: Bromo‐trimethylsilane
^Cat^
: catalytic subunit
Cdk1: cyclin‐dependent kinase 1
Ctrl.: control
DCM: dichloromethane
DDA: data‐dependent acquisition
DiFMUP: 6,8‐Difluoro‐4‐Methylumbelliferyl Phosphate
DIPEA: N,N‐Diisopropylethylamine
DMF: N,N‐dimethylformamide
DMSO: dimethyl sulfoxide
DTT: dithiothreitol
EDT: ethane‐1,2‐dithiol
Et_2_O: diethlyether
FDR: false discovery rate
Fmoc: fluorenylmethyloxycarbonyl
Fmoc‐PEG_2_
: {2‐[2‐(Fmoc‐amino)ethoxy]ethoxy}acetic acid
Fmoc‐Pfa‐Et_2_
: (2 s)‐4‐(diethodxyphosphoryl)‐2‐({[(9 h‐fluoren‐9‐yl)methoxy]carbonyl}amino)‐4,4‐difluorobutanoic acid
HBTU: O‐benzotriazole‐*N*,*N*,*N′*,*N′‐tetramethyluronium*hexafluorophosphate
HCD: higher energy collision‐induced dissociation
HOBt: *N*‐hydroxybenzotriazole hydrate
HSP: heat shock protein
LFQ: Label‐Free Quantification
LiAc: lithium acetate
MALDI: matrix‐assisted laser desorption/ionization
maxIT: maximum injection time
MS: mass spectrometry
NCE: normalized collision energy
NMM: N‐methylmorpholine
NT: no tag
PEG: polyethylene glycol
Pfa: phosphono‐difluoromethyl‐alanine
Pma: phosphono‐methyl‐alanine
PMSF: phenylmethylsulfonyl fluoride
PP1: protein phosphatases 1
PP2A: protein phosphatase 2A
PP4: protein phosphatase 4
PPP: phosphoprotein phosphatase family
Prep‐HPLC: preparative high‐performance liquid chromatography system
ProtA: protein A
pSer/pS: phosphoserine
PSM: peptide spectrum match
pT: phosphothreonine
PVDF: polyvinylidene difluoride
^Reg^
: regulatory subunit
^Scaf^
: scaffolding subunit
SLiM: short linear motif
SPPS: solid phase peptide synthesis
TBS‐T: Tris‐buffered saline containing 0,1% Tween‐20
TFA: trifluoroacetic acid
TIPS: triisopropylsilane
TOM: translocase of the mitochondrial outer membrane
w/o: without
wt: wild type

## Introduction

Most mitochondrial proteins are encoded in the nucleus and imported and sorted into the different mitochondrial sub‐compartments post‐translationally. It was previously believed that the mere presence of pre‐proteins is sufficient to induce import and sorting. However, a pioneering study in 2011 revealed that many components of the import machinery possess phosphorylation sites, some of which play a significant role in the regulation of import [1]. While kinases responsible for phosphorylating several of these sites have already been identified [1, 2, 3], the counteracting phosphatases remain elusive.

**Fig. 1:**
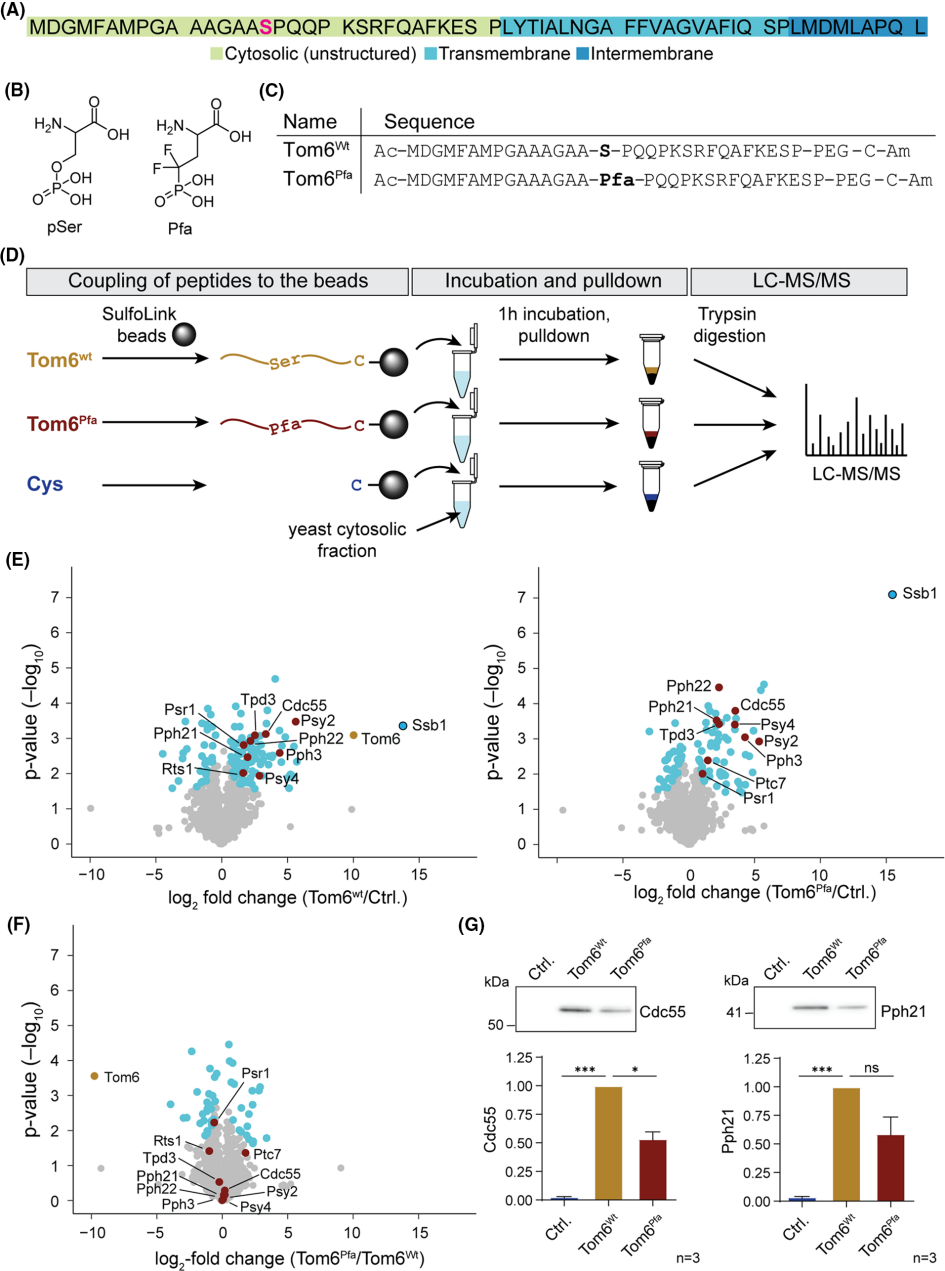
Protein enrichment with Tom6 trap‐peptides. (A) Yeast Tom6 full‐length sequence with the phosphorylation site at serine 16 shown in red, and structural elements colored as indicated. (B) Structure of pSer and the phosphomimetic phosphono‐difluoromethyl‐alanine (Pfa). (C) Tom6 peptide sequences containing the full cytosolic domain as wild‐type (wt) sequence or with the pSer mimetic Pfa. Peptides were *N*‐ and *C*‐terminally acetylated (Ac) and amidated (Am), respectively. Between the Tom6 sequence and the cysteine for coupling to SulfoLink beads, a polyethylene glycol (PEG) linker was included. (D) Workflow to identify proteins binding to the Tom6 trap‐peptides compared to the control beads blocked with cysteine. LC–MS/MS: Liquid chromatography‐based tandem mass spectrometry. (E) Proteins enriched with the Tom6 trap‐peptides. Volcano plots showing the enriched proteins when comparing the control with cysteine (Ctrl.) and the two trap‐peptides Tom6^wt^ and Tom6^Pfa^. Blue dots represent significantly enriched proteins (significance threshold: FDR = 0.05, s0 = 0.5), the outlined blue dot represents the most enriched protein (Ssb1), the yellow dot represents Tom6, and significantly enriched serine/threonine catalytic and regulatory phosphatase subunits are shown in red. Proteomics data can be found in Table S1. (F) Volcano plot comparing the proteins enriched between both peptides (Tom6^wt^ and Tom6^Pfa^). Blue dots represent significantly enriched proteins (significance threshold: FDR = 0.05, s0 = 0.5) and the red ones are the significantly enriched serine/threonine catalytic and regulatory phosphatase subunits from the volcano plots in E. Proteomics data can be found in Table S1. (G) Comparison of the PP2A catalytic subunit Pph21 and the regulatory subunit Cdc55 enrichment by pulldown with both Tom6 peptides (wt and Pfa) and the control. The amounts of bound PP2A subunits were analyzed by western blot. Western blots containing the input and a Coomassie‐stained gel of the pull‐down are included in Fig. S1C. For the statistical analysis of the quantified blots, a one‐way ANOVA followed by a Tukey's multiple comparisons test was performed. Error bars of the graphs represent the standard error of the mean. ns: *P* > 0.05, *P ≤ 0.05, ***P ≤ 0.001.

The Translocase of the mitochondrial Outer Membrane (TOM) complex is the main entry gate for mitochondrial precursor proteins. It is composed of the core proteins Tom40 (the import channel), Tom22, Tom5, Tom6, and Tom7 to which the peripheral import receptors Tom20 and Tom70 loosely associate. Tom6, a small transmembrane protein, plays a role in assembling trimeric versions of the complex together with Por1, a voltage‐dependent anion channel [4, 5]. Within its cytosolic domain, Tom6 contains a phosphorylation site at Ser16 (Fig. 1A). Harbauer *et al*. demonstrated that phosphorylation at this site is catalyzed by cyclin‐dependent kinase 1 (Cdk1) [2]. During the M phase of the cell cycle, Cdk1 is activated by Clb3 cyclin, resulting in an increased number of phosphorylated Tom6 precursor proteins. This leads to enhanced import of Tom6, increasing the assembly of TOM complexes, thereby facilitating an increased protein import rate to meet the elevated energy demands during cell cycle progression. Mass spectrometry (MS) data published by Godfrey *et al*. also detected a rapid increase of Tom6 phosphorylation following the release of yeast cells from the G1 phase [6]. The levels remained stable until the end of the experiment at 90 min after release from G1 phase.

Linking phosphatases to their target sites remains a considerable challenge. Yeast cells express 127 kinases, of which 90% are evolutionarily conserved and predominantly regulated through phosphorylation [7]. In contrast, only 43 phosphatases with diverse evolutionary backgrounds counteract kinase activity. Some of these phosphatases are multidomain enzymes. Others are regulated by the formation of holoenzymes through the interaction of the catalytic subunit (^cat^) with regulatory proteins (^reg^) and/or scaffolding proteins (^scaf^), like the phosphoserine/threonine‐specific protein phosphatases‐1 (PP1), ‐4 (PP4) and ‐2A (PP2A), increasing the complexity of their interactions. In addition, many phosphatases interact transiently with their substrates, making them difficult to identify [8, 9, 10].

A hydrolysis‐stable phosphoserine (pSer) mimetic could potentially enable stable binding of phosphatases to their specific phosphorylation sites. While glutamic acid or aspartic acid are often used as pSer mimetics due to their ease of incorporation, their different structures and charges compared to a phosphate group render them largely ineffective for binding phosphatases [9, 11]. Thus, other non‐hydrolysable pSer mimetics containing a phosphonate group have been developed, which better resemble pSer chemically. Two mimetics, phosphono‐methyl‐alanine (Pma) and phosphono‐difluoromethyl‐alanine (Pfa), are available, in which the bridging oxygen is replaced by a methyl or a CF_2_ group, respectively (Fig. 1B) [12, 13, 14]. The CF_2_ group in Pfa closely resembles pSer due to its isopolarity, and its estimated pKa_2_ value (5.1) is closer to that of pSer (5.7) than that of Pma (7.1) [15, 16]. Replacement of pSer by Pfa has been successfully applied for the development of phospho‐specific antibodies [17] and used in a potent 14–3‐3 protein inhibitor prodrug [18] and in BRCA1 and Cdc14 inhibitors [19, 20]. While Pma has been used in bait proteins, no phosphatases were found to bind to these baits; only other interacting proteins were detected that bind in a phosphorylation‐dependent manner [21, 22]. Pfa, predicted to be a better mimetic, might bind and trap phosphatases more efficiently than Pma. Therefore, we used synthetic peptides referred to as “trap‐peptides” containing the amino acids surrounding the phosphorylation site of interest and either Pfa or the wild‐type Ser to capture phosphatases that target Ser16 of Tom6 (Fig. 1C). Synthetic peptides are often unstructured and would not necessarily fold to the structure the sequence would have if found in a folded region of a protein. Nevertheless, many phosphorylation sites, like pSer16 on Tom6, are located in disordered regions of proteins for which trap‐peptides are ideal to model this unstructured state.

Through this approach, PP4 and PP2A emerged as two candidate phosphatases that interact with Tom6. Remarkably, they were identified as full holoenzymes, with both binding to Tom6 through a regulatory subunit independently of the phosphorylation status of Ser16, including the incorporation of Pfa. Subsequent *in vitro* assays confirmed PP2A/Cdc55^reg^ as the first TOM phosphatase that dephosphorylates Tom6 pSer16; whereas PP4/Psy2^reg^, in spite of interacting with Tom6, did not hydrolyze this phosphorylation site.

## Results

### Tom6 trap‐peptides enrich PP4 and PP2A as full holoenzymes

The small size of Tom6 makes it an ideal candidate for trap‐peptide design (Fig. 1A), as the entire cytosolic domain, containing the phosphorylation site of interest (Ser16), is unstructured like the trap‐peptides, offering a similar interaction space. The small size was also beneficial for solid phase peptide synthesis (SPPS), that was efficient particularly to include the non‐natural amino acid Pfa. Two different Tom6 trap‐peptides were designed for pull‐down experiments (Fig. 1B). Both contain the full cytosolic domain, a *C*‐terminal polyethylene glycol (PEG) linker as a spacer between the peptides and the beads, and a cysteine for coupling to SulfoLink beads. The first peptide comprised the wild‐type (wt) Tom6 sequence, while the second incorporated the pSer mimetic Pfa at the Ser16 position (Fig. 1C).

The trap‐peptides were coupled to SulfoLink beads and incubated with cytosolic fractions from yeast grown in non‐fermentable medium (YPG, glycerol as carbon source). As a control, SulfoLink beads were blocked with cysteine and subjected to the same treatment as the trap‐peptides. After incubation, interacting proteins were enriched by bead pull‐down (Fig. 1D). Mass spectrometry (MS) analysis of the samples revealed several significantly enriched phosphatases compared to the negative cysteine control (Fig. 1E; Fig. S1A, Tables [Link], [Link]). However, when comparing the proteins enriched between both trap‐peptides, no phosphatase was significantly enriched by the Tom6^Pfa^ trap‐peptide (Fig. 1F, Table S4). For both peptide samples, Ssb1, a member of the heat shock protein 70 family (HSP70) associated with ribosomes [23], was the most abundantly enriched protein.

The Tom6 cytosolic peptide, that is the bait, was identified as significantly enriched only in samples with the Tom6^wt^ peptide. This is due to the mass of Pfa not being included in the mass spectrometric database search; thus, no peptides containing position 16 were identified in the samples containing Tom6^Pfa^ (Fig. S1B). However, one peptide at the *C*‐terminal end of the cytosolic domain did not entail Ser16 (SRFQAFK) and could therefore be detected in Tom6^wt^ as well as Tom6^Pfa^ samples. This peptide showed a similar mass spectrometric intensity across samples, suggesting a very comparable trap‐peptide concentration between Tom6^wt^ and Tom6^Pfa^ and a release from the beads (Fig. S1B).

Several serine/threonine‐specific phosphatases and their regulatory subunits were significantly enriched with both trap‐peptides (Fig. 1E, indicated in red; Tables S2 and S3). Notably, PP4 and PP2A were detected as full trimeric holoenzymes. For PP2A, which usually consists of a catalytic subunit, a regulatory subunit, and a scaffolding subunit, two catalytic subunits (Pph21^cat^ and Pph22^cat^), the regulatory subunit Cdc55^reg^ (homolog of human B55), and the scaffolding protein Tpd3^scaf^ were identified [24, 25]. The regulatory subunit Rts1^reg^ (homolog of human B56) was only enriched, and to a small extent, in the Tom6^wt^ peptide samples. For PP4, which usually consists of the catalytic subunit with one or two regulatory subunits, we identified the catalytic subunit Pph3^cat^ and the two regulatory subunits Psy2^reg^ and Psy4^reg^ [26, 27]. Although Ptc7 and Psr1 were significantly enriched with one or both of the trap‐peptides, they were not followed up on as potential phosphatase candidates as they had lower *P*‐values than many of the PP4 and PP2A subunits. Here, the binding of the phosphatases did not depend on the presence of Pfa, suggesting that the interaction is likely not mediated through the interaction of the phosphorylation site with the catalytic subunit of the holoenzymes.

To analyze the binding of phosphatase subunits in a quantitative manner to both peptides, the pulldowns were repeated in triplicates and analyzed by western blot. Due to the unavailability of antibodies against yeast PP4 subunits, we focused on two PP2A subunits, namely Cdc55^reg^ and Pph21^cat^ (Fig. 1G). Both proteins bound less well to the Tom6 peptide containing the Pfa. This indicates that either Pfa is not a suitable mimetic for pSer in this case or that PP2A preferably binds to the unphosphorylated Tom6.

### PP2A and PP4 interact with Tom6 through their regulatory subunits

**Fig. 2:**
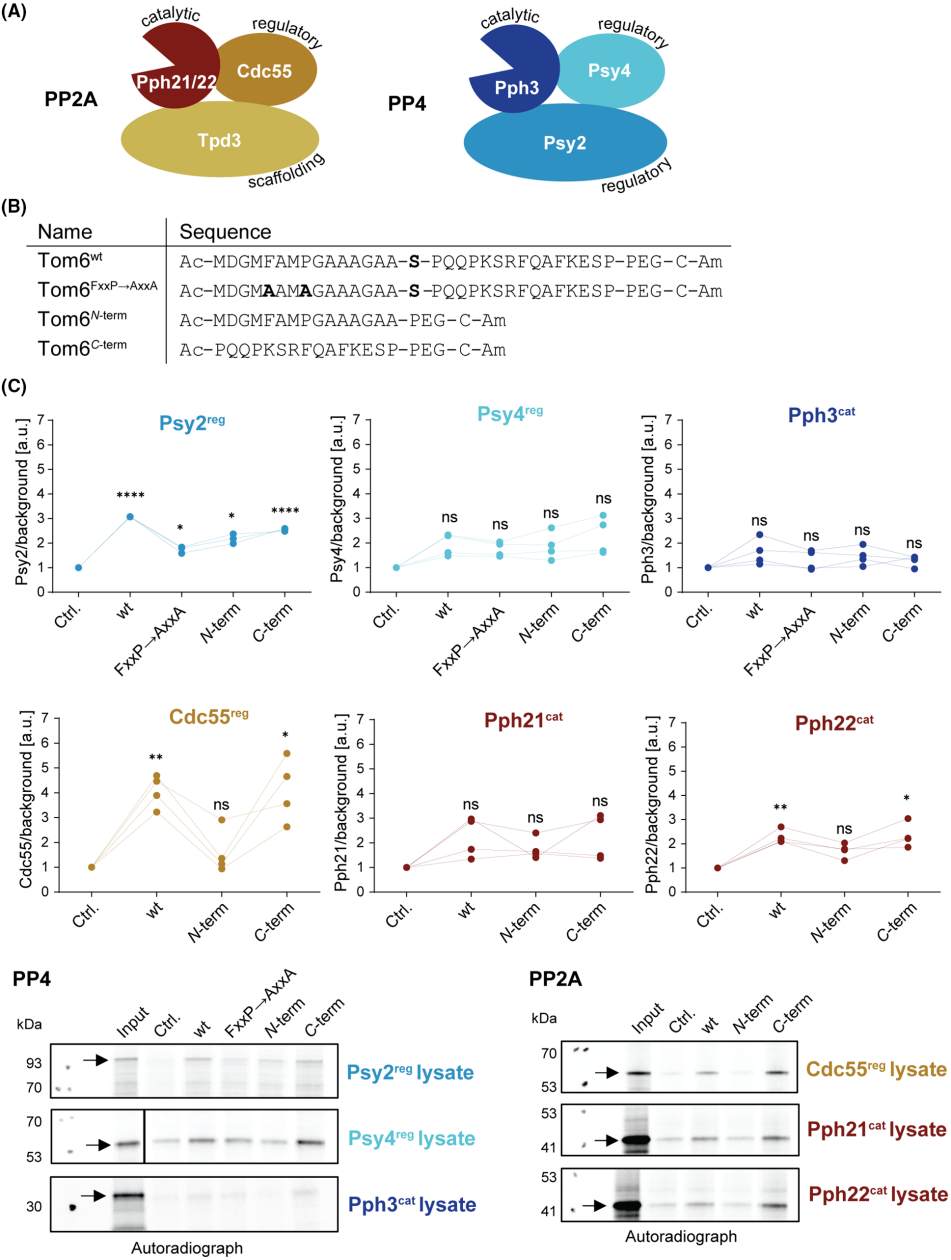
Interaction between Tom6 and individual subunits of PP4 and PP2A. (A) PP4 (containing the catalytic subunit and two regulatory subunits) and PP2A holoenzyme (containing one of the two catalytic subunits, one regulatory and one scaffolding subunit) compositions that were significantly enriched with Tom6 trap‐peptides. The subunits were translated in rabbit reticulocyte lysate in the presence of ^35^S‐methionine. (B) Tom6 peptides for the pull‐down assays with the phosphatase subunits contain the full‐length Tom6 cytosolic domain either wild‐type (wt) or with a mutated Psy2 binding motif (FxxP→AxxA). Additionally, two shorter peptides of the sequence *N*‐ or *C*‐terminally of the phosphorylation site were synthesized. All peptides were *N*‐ and *C*‐terminally acetylated (Ac) and amidated (Am) respectively and contain a polyethylene glycol (PEG) linker. (C) Pull‐downs were performed by incubating Sulfolink beads loaded with the different peptides or cysteine as control (Ctrl.) with the individual ^35^S‐methionine‐labeled phosphatase subunits for 1 h. Autoradiographs of the pull‐downs were quantified and normalized to the background of the individual lanes. The arrows indicate the band of the overexpressed protein. *n* = 4 for all subunits except for Psy2^reg^ for which *n* = 3. For the statistical analysis, a one‐way ANOVA followed by a Tukey's multiple comparisons test was performed. *P*‐values indicated represent the comparison to the control, ns: *P* > 0.05, *P ≤ 0.05, **P ≤ 0.01, ****P ≤ 0.0001.

To test the hypothesis that Tom6 interacts with a regulatory protein of the holoenzyme rather than the catalytic subunit, we performed binding studies with radiolabeled proteins. Individual phosphatase subunits were translated *in vitro* using rabbit reticulocyte lysate in the presence of ^35^S methionine (Fig. 2A). For PP4, the subunits Psy2^reg^, Psy4^reg^, and Pph3^cat^ were labeled, and for PP2A, Pph21^cat^, Pph22^cat^, and Cdc55^reg^ could be successfully labeled. In addition to the Tom6^wt^ peptide used in previous pull‐down experiments, further peptides were included to narrow down the binding site and to determine if the presence of the phosphorylation site is required for binding (Fig. 2B). The binding motif of the human PP4 regulatory subunit PPP4R3A/B, which corresponds to Psy2^reg^ in yeast, has been identified to bind to an FxxP motif [28]. Due to the high conservation between yeast and human PP4, this binding motif is also expected to be present in yeast. Indeed, the cytosolic domain of Tom6 contains an FxxP motif. To assess whether this motif is necessary for interaction with Psy2^reg^, we synthesized a peptide in which the FxxP motif is mutated to alanine (AxxA) (Fig. 2B). We also included two shorter peptides from the regions *N*‐ or *C*‐terminal to Ser16 to narrow down the binding site and to determine if Ser16 itself plays a role in the interaction.

The radiolabeled subunits were incubated with the Tom6 peptides coupled to SulfoLink beads, and pull‐downs were carried out to enrich the subunits (Fig. 2C, Fig. S2). From the PP4 subunits, only Psy2^reg^ significantly bound to the peptides compared to the negative control (cysteine). Since Psy4^reg^ and Pph3^cat^ were found in the pulldown from the cell lysates (Fig. 1E), this suggests that the full holoenzyme consisting of Psy2^reg^, Psy4^reg^, and Pph3^cat^ binds to Tom6 via Psy2^reg^. Reduced binding to the other peptides compared to the Tom6^wt^ peptide was observed with the mutated FxxP motif and the *N*‐terminal peptide. Yet, the *N*‐terminal peptide contains the FxxP motif, which was expected to mediate the interaction. Instead, the *C*‐terminal peptide showed almost as strong a binding to Psy2^reg^ as the full‐length peptide (the significant differences between the different peptides are shown in Fig. S2). Therefore, the motif appears to only play a minor role or be negligible for Psy2^reg^ binding.

Similarly, for PP2A, the regulatory subunit Cdc55^reg^ significantly bound to the Tom6^wt^ and the *C*‐terminal peptides (Fig. 2C). Therefore, the *C* terminus of the cytosolic domain from Tom6 contains the recognition site for binding to Cdc55^reg^ of the PP2A/Cdc55^reg^/Tpd3^scaf^ holoenzyme, as identified in the pulldown from yeast lysates (Fig. 1E). Among the two catalytic subunits, only Pph22^cat^ bound significantly to the Tom6^wt^ and *C*‐terminal peptides; although the interaction was weaker than the one observed with Cdc55^reg^. This could suggest that the catalytic subunit also takes part in the binding event with Tom6, although to a lesser extent than Cdc55^reg^.

These results suggest that both the PP4 and PP2A holoenzymes interact with Tom6 with their respective regulatory subunits, Psy2^reg^ and Cdc55^reg^, and that this binding appears to be independent of the presence of Pfa.

### Purified PP4 holoenzyme did not dephosphorylate Tom6 at serine 16

**Fig. 3:**
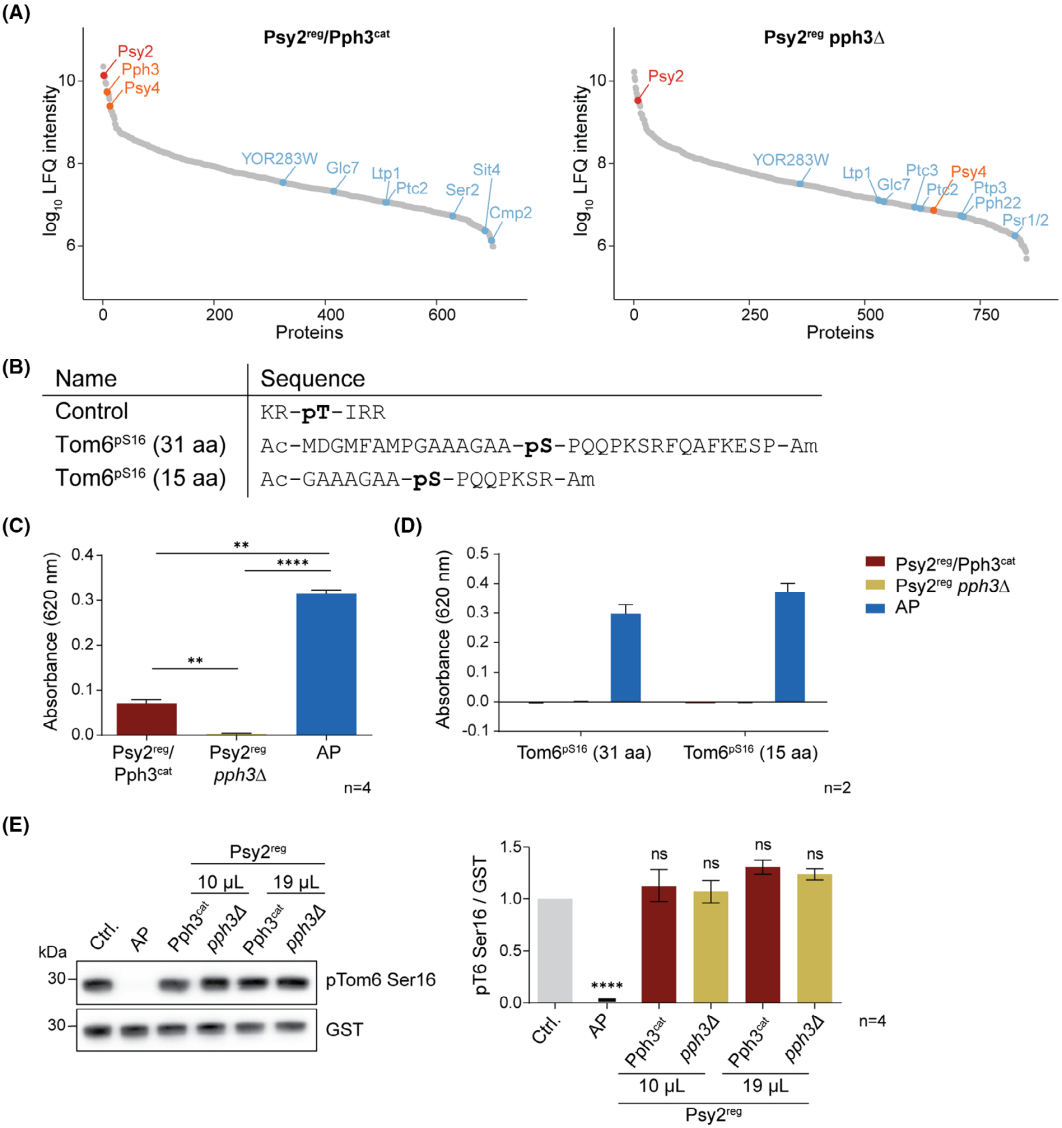
Phosphatase activity of PP4/Psy2 holoenzyme (Psy2^reg^/Pph3^cat^) purified through tagged Psy2^reg^ from yeast. (A) PP4 holoenzyme was purified through a pull‐down of tagged Psy2^reg^ from yeast (Pys2^reg^/Pph3^cat^). As a control, Psy2^reg^ was tagged and purified in a yeast strain missing the catalytic subunit Pph3^cat^ (Psy2^reg^ *pph3*Δ). The purified products were analyzed by mass spectrometry and the identified proteins sorted by LFQ intensity. The bait, Psy2^reg^, is labeled in red, other PP4 subunits in orange, and all other found catalytic phosphatase subunits in blue. Proteomics data can be found in Table S5. (B) Peptides containing phosphoserine or phosphothreonine (pS, pT) to test the phosphatase activity of Psy2^reg^/Pph3^cat^. (C) The control peptide was incubated with Psy2^reg^ purified either from wild‐type yeast (Psy2^reg^/Pph3^cat^) or from Pph3 knock‐out yeast (Psy2^reg^ *pph3*Δ) for 1 h at 30 °C. As a positive control, alkaline phosphatase (AP) was included. The amount of free phosphate was quantified by the addition of malachite green. (D) Malachite green assay with the pS‐containing Tom6 peptides. The same reaction conditions as with the control peptide were applied. (E) Recombinant Tom6pSer^16^‐GST was incubated with 10 or 19 μL of the purified Psy2^reg^/Pph3^cat^ or Psy2^reg^ *pph3*Δ for 1 h at 30 °C. As a negative control, no phosphatase was added (Ctrl.) and for the positive control, alkaline phosphatase (AP). Dephosphorylation was monitored with pTom6 Ser16 and GST antibodies. Error bars of all graphs represent the standard error of the mean. For the statistical analysis, a one‐way ANOVA followed by a Tukey's multiple comparisons test was performed. *P*‐values indicated represent the comparison to the control, ns: *P* > 0.05, **P ≤ 0.01, ****P ≤ 0.0001.

To further assess PP4 as a phosphatase targeting serine 16 of Tom6, we performed *in vitro* dephosphorylation assays. To isolate the PP4 holoenzyme from yeast, Psy2^reg^ was tagged with protein A (ProtA) via homologous recombination in wt and *pph3Δ* yeast cells. The knock‐out strain missing the PP4 catalytic subunit Pph3^cat^ was included as a control to confirm that any observed phosphatase activity stems from Pph3^cat^ and not from other potentially enriched catalytic phosphatase subunits. After purification following standard protocols [29] the products were analyzed by western blot (Fig. S3A) as well as mass spectrometry (Fig. 3A, Table S5, Fig. S3B). The Psy2^reg^ sample purified from wt yeast showed enrichment of the bait protein Psy2^reg^ as well as PP4 subunits Pph3^cat^ and Psy4^reg^. This holoenzyme will be referred to here as Psy2^reg^/Pph3^cat^. Meanwhile, the Psy2^reg^ sample isolated from *pph3Δ* (Psy2^reg^ *pph3Δ*) did not contain Pph3^cat^ as expected and low amounts of Psy4^reg^. Additional phosphatases were identified by MS for both samples but in very low amounts (Fig. 3A). Therefore, any phosphatase activity that would be detected in the assay should be due to Pph3^cat^ from wt yeast.

As a positive control to assess the activity of purified Psy2^reg^/Pph3^cat^, we used a phosphothreonine containing peptide known to be dephosphorylated by PP4 [29] (Fig. 3B). Only Psy2^reg^/Pph3^cat^ purified from wt yeast was able to dephosphorylate the control peptide (Fig. 3C). Next, peptides based on the Tom6 sequence in two different lengths containing pSer at position 16 were synthesized (Fig. 3B). Neither peptide was dephosphorylated by Psy2^reg^/Pph3^cat^ after incubation for 1 h at 30 °C (Fig. 3D). However, when tested with alkaline phosphatase, both peptides were dephosphorylated, confirming again the functionality of the peptides and assay.

As an alternative approach, the cytosolic domain of Tom6 was recombinantly expressed with a GST tag in *E. coli* (Fig. S3C) [2]. To incorporate the pSer, an amber stop codon was introduced, and pSer was incorporated using the amber suppression technology [22]. The correct incorporation was confirmed using a Tom6 pSer16‐specific antibody (Fig. S3D). However, the pSer‐containing Tom6 was also not dephosphorylated by Psy2^reg^/Pph3^cat^ during co‐incubation for 1 h at 30 °C (Fig. 3E).

In summary, while the PP4/Psy2^reg^ holoenzyme binds to Tom6, the *in vitro* dephosphorylation assays using purified PP4/Psy2^reg^ holoenzyme (in form of yeast Psy2^reg^/Pph3^cat^/Psy4^reg^) and Tom6 proteins do not support a role for PP4/Psy2^reg^ in dephosphorylating serine 16 of Tom6.

### The PP2A holoenzyme dephosphorylated Tom6 at serine 16

**Fig. 4:**
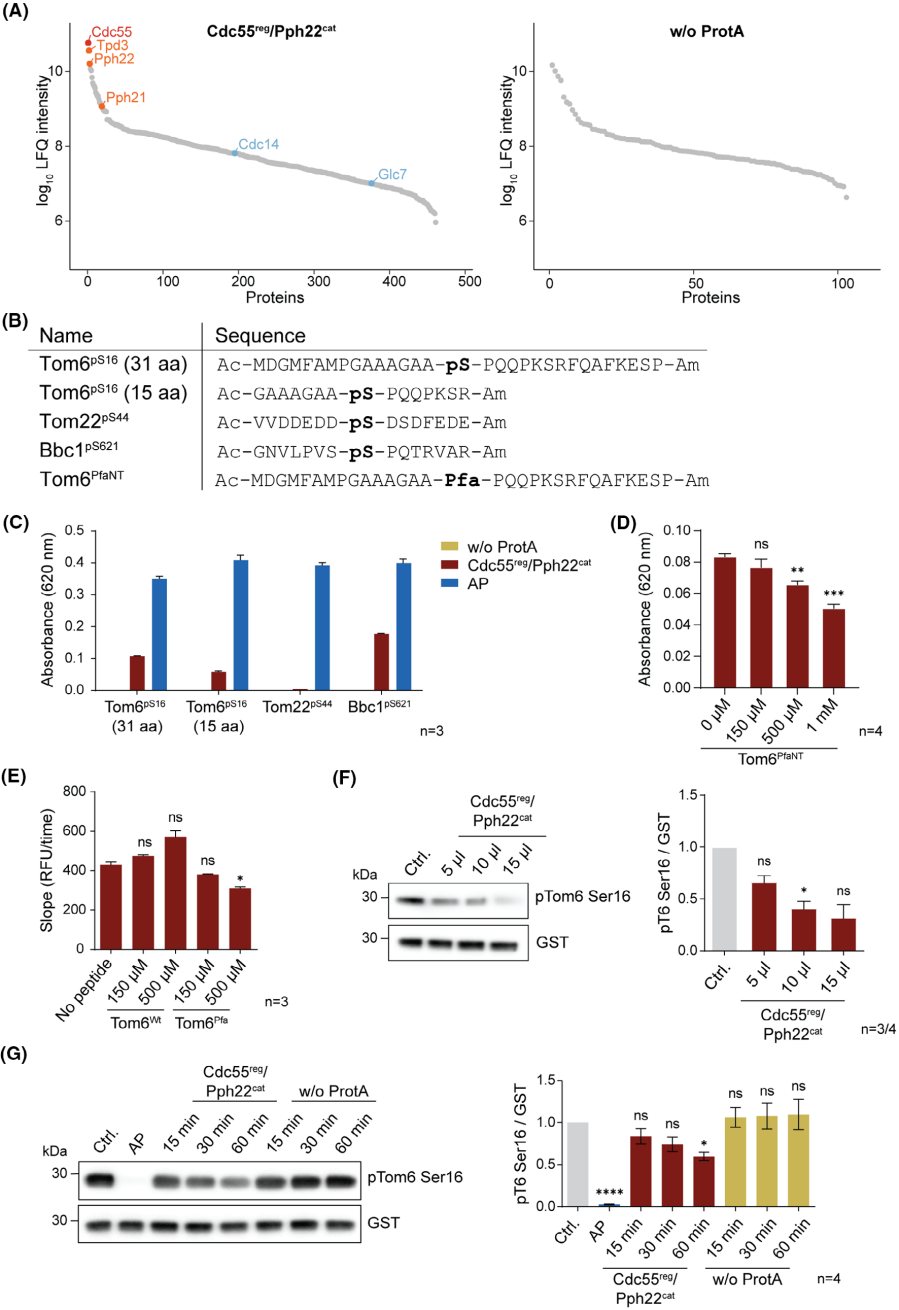
PP2A/Cdc55 holoenzyme (Cdc55^reg^/Pph22^cat^) purified through tagged Cdc55^reg^ from yeast dephosphorylates Tom6 pSer16. (A) PP2A holoenzyme was purified through a pull‐down of tagged Cdc55^reg^ from yeast (Cdc55^reg^/Pph22^cat^). As a control, the purification was also performed in a yeast strain without tagged protein (w/o ProtA). The purified products were analyzed by mass spectrometry, and the identified proteins were sorted by LFQ intensity. The bait, Cdc55^reg^, is labeled in red, other PP2A subunits in orange, and all other found catalytic phosphatase subunits in blue. Proteomics data can be found in Table S5. (B) Peptides containing phosphoserine (pS) to test the phosphatase activity of Cdc55^reg^/Pph22^cat^ and a non‐tagged (NT) Tom6 peptide containing Pfa. Peptides were *N*‐ and *C*‐terminally acetylated (Ac) and amidated (Am) respectively. (C) Malachite green assay with the pS‐containing peptides and as phosphatases the purified Cdc55^reg^/Pph22^cat^, the negative control without ProtA, and alkaline phosphatase (AP). Peptides and phosphatases were incubated for 1 h at 30 °C. (D) Malachite green competition assay between Tom6^pSer16^ and Tom6^PfaNT^. Cdc55^reg^/Pph22^cat^ was preincubated with the indicated concentrations of Tom6^PfaNT^ for 10 min at room temperature before adding Tom6^pSer16^ and incubating for 1 h at 30 °C. (E) Cdc55^reg^/Pph22^cat^ was preincubated with Tom6^wt^ or Tom6^Pfa^ (Fig. 1C) or no peptide for 10 min before adding DiFMUP and monitoring its dephosphorylation to DiFMU. RFU: relative fluorescence unit. (F) Recombinant Tom6pSer^16^‐GST was incubated with different amounts (5, 10 or 15 μL) of Cdc55^reg^/Pph22^cat^ or no phosphatase as control (Ctrl.) for 1 h at 30 °C. The dephosphorylation was monitored by western blot. (G) Incubation of recombinant Tom6pSer^16^‐GST with 10 μL Cdc55^reg^/Pph22^cat^ or the negative control w/o ProtA for 15, 30, or 60 min at 30 °C. As negative control, no phosphatase was added (Ctrl.) and for the positive control, alkaline phosphatase (AP). The dephosphorylation of Tom6pSer‐GST was monitored by western blot with pTom6 Ser16 and GST antibodies. For all graphs, the error bars represent the standard error of the mean. For the statistical analysis, a one‐way ANOVA followed by a Tukey's multiple comparisons test was performed. *P*‐values indicated represent the comparison to the control, ns: *P* > 0.05, *P ≤ 0.05, **P ≤ 0.01, ***P ≤ 0.001.

While PP4 did not exhibit dephosphorylation activity towards Tom6 *in vitro*, we next tested the PP2A holoenzyme. The regulatory subunit Cdc55^reg^ was tagged with ProtA via homologous recombination in wild‐type yeast cells [30]. For comparison with a negative control, PP2A purification was performed in parallel with a cell lysate containing tagged and non‐tagged Cdc55^reg^, since the knock‐out of both catalytic subunits (in analogy to the PP4 experiments), Pph21^cat^ and Pph22^cat^, results in severe growth defects [31]. Enrichment of Cdc55^reg^, the catalytic subunit Pph21^cat^, and the scaffolding protein Tpd3^scaf^ was confirmed by western blot (Fig. S4A,B). To further investigate the composition of the holoenzyme, samples were again analyzed by mass spectrometry (Fig. 4A, Table S5, Fig. S4C). In the negative control, no PP2A subunits (Cdc55^reg^, Tpd3^scaf^, Pph21^cat^, or Pph22^cat^) or other catalytic phosphatase subunits were detected. In the Cdc55^reg^‐tagged sample, the three most enriched proteins were Cdc55^reg^, Tpd3^scaf^, and Pph22^cat^, matching the holoenzyme composition found in the Tom6 pull‐down from yeast cytosolic fractions (Fig. 1E). This PP2A holoenzyme purified by tagging Cdc55^reg^ will be referred to here as Cdc55^reg^/Pph22^cat^.

Next, different peptides containing a pSer were synthesized to test the ability of the PP2A holoenzyme to dephosphorylate them (Fig. 4B). As for PP4, two versions of the Tom6 peptide were used. Additionally, a Bbc1 site, previously found to be hyperphosphorylated in Cdc55^reg^ knock‐out yeast [6], served as a positive control. As a negative control, we included a phosphorylation site in Tom22, which is surrounded by acidic amino acids known to be disfavored by human PP2A/B55 [32]. Cdc55^reg^/Pph22^cat^ was able to dephosphorylate Bbc1 and both Tom6 peptides (Fig. 4C). Notably, a slight preference for the longer Tom6 peptide was observed, suggesting that the omitted amino acids may be involved in the interaction of Cdc55^reg^ with Tom6. As anticipated, Cdc55^reg^/Pph22^cat^ did not dephosphorylate the Tom22 peptide, while all peptides were dephosphorylated by the positive control with alkaline phosphatase. To study the ability of a Pfa‐containing peptide to compete with the Tom6^pS16^ one, we synthesized Tom6^PfaNT^ containing the Tom6 cytosolic domain with an Pfa at position 16 but no tag (NT) (Fig. 4B). Cdc55^reg^/Pph22^cat^ was preincubated with different Tom6^PfaNT^ amounts before adding the same amount of Tom6^pS16^ substrate to all conditions. Increasing Tom6^PfaNT^ concentrations led to reduced Tom6^pS16^ dephosphorylation, showing a competitive behavior of Tom6^PfaNT^ to Tom6^pS16^ (Fig. 4D). However, an excess of Tom6^PfaNT^ was necessary to significantly reduce the dephosphorylation, indicating that the addition of the Pfa to the peptide did not lead to stronger binding to Cdc55^reg^/Pph22^cat^ than the phosphorylated peptide.

The ability of Pfa to insert into the catalytic pocket of PP2A remains to be elucidated. To this end, we compared the ability of Tom6^Wt^ and Tom6^Pfa^ (Fig. 1C) to block the catalytic pocket of Cdc55^reg^/Pph22^cat^. 6,8‐difluoro‐4‐methylumbelliferyl phosphate (DiFMUP) was used as a substrate for this assay. The reaction product DiFMU obtained by dephosphorylation is a fluorophore, which can be quantified to assess the activity of the tested phosphatase. Due to its small size, it should not interfere with the binding of the peptides to the regulatory subunit Cdc55. While Tom6^Wt^ did not prevent DiFMUP dephosphorylation, the addition of Tom6^Pfa^ decreased the dephosphorylation, supporting again competition with the substrate in the active site (Fig. 4E). The decrease was significant when adding 500 μm Tom6^Pfa^. However, it only partially reduced the activity of Cdc55^reg^/Pph22^cat^. This suggests that Pfa is not able to strongly bind in an orientation occupying the catalytic pocket of PP2A, making it a weak inhibitor. Thus, at least for PP2A, Pfa is not a pSer mimetic which readily inserts and remains in the catalytic cleft.

Finally, contrary to the finding with the PP4 holoenzyme, recombinantly expressed Tom6pSer^16^‐GST was dephosphorylated by Cdc55^reg^/Pph22^cat^ (Fig. 4F,G), strongly supporting that pSer16 of Tom6 is a substrate of this holoenzyme. The complexity of the purified holoenzyme only allows for an approximate concentration prediction of the fully functional trimeric complex. To address this caveat, different amounts of purified Cdc55^reg^/Pph22^cat^ were compared in their ability to dephosphorylate Tom6 pSer16. A concentration‐dependent dephosphorylation trend of Tom6pSer^16^‐GST was observed after 1 h incubation (Fig. 4F), corroborating that the PP2A holophosphatase can specifically dephosphorylate Tom6.

Together, our biochemical data, based on trap‐peptides and activity validation, strongly supports that the PP2A/Cdc55^reg^ holoenzyme (in form of yeast Cdc55^reg^, Tpd3^scaf^, and Pph22^cat^) dephosphorylates Tom6 at pSer16.

## Discussion

To identify phosphatase(s) that can bind to and dephosphorylate Ser16 of Tom6, we used trap‐peptides. They contain the cytosolic domain of Tom6 with either the wt serine or the non‐hydrolizable pSer mimetic Pfa. Both were coupled to beads and used for a pull‐down to enrich interacting proteins from yeast cytosolic fractions. Both trap‐peptides significantly enriched several phosphatases compared to the negative control. Of the identified phosphatases, PP4 and PP2A were most enriched, and both were found as full heterotrimeric holoenzymes. The catalytic subunits of the phosphatases are very similar, as Pph3^cat^ can take over at least the essential functions of Pph21^cat^ and Pph22^cat^ if forced [31].

Even though the Pfa was included as a pSer mimetic, we observed that its presence reduced the binding of PP2A and that in *in vitro* assays, in spite of the peptide binding to the holoenzyme, Pfa did not block the active site of PP2A very effectively. Our results point to Pfa not being a good pSer mimetic for PP2A. Potentially, the added CF_2_ group prevents interaction and insertion into the catalytic pocket as it is bulkier than oxygen. A previous study by DeMarco *et al*. found that a seven amino acid long peptide based on the ideal substrate sequence for Cdc14 containing Pfa inhibits Cdc14 activity [20]. Contrary to kinases, phosphatases evolved from different evolutionary backgrounds resulting in different catalytic mechanisms [33]. The majority of phosphatases dephosphorylating pTyr or dual specificity ones dephosphorylating pTyr, pSer, and pThr rely on a cysteine in their catalytic cleft for the cleavage of the phosphate group [34]. This is also the case for Cdc14, a dual specificity phosphatase with a preference for pSer‐Pro motifs [20]. Meanwhile, members of the phosphoprotein phosphatase family (PPPs; PP1, PP2A, Calcineurin, PP4, PP5, PP6, PP7) rely on divalent cations in the catalytic cleft instead of a cysteine [35]. Even though we only showed this for the catalytic subunit of PP2A, it seems unlikely that Pfa stably binds to the catalytic cleft of other members of the PPP family. Thus, we hypothesize that the efficiency of Pfa as a pSer mimetic to bind/block the catalytic pocket might depend on the catalytic mechanism of the phosphatase. Until now, pTyr and pSer mimetics containing a CF_2_ group instead of the oxygen (F_2_Pmp and Pfa) were only shown to bind the catalytic cleft of phosphatases relying on a cysteine for the cleavage of the phosphate group [20, 36, 37, 38, 39, 40]. Further studies are necessary to validate this hypothesis.

The protein with the highest fold change for both Tom6 trap‐peptides compared to the negative control was Ssb1, a member of the HSP70 family. According to the CRAPome database, HSPs often bind non‐specifically in pull‐down experiments [41]. However, Ssb proteins associate with nascent polypeptide chains to prevent aggregation until they are imported into mitochondria [42, 43, 44], and some HSP members directly interact with yeast and human import receptors during the import process [5, 45, 46]. Thus, the interaction of Ssb1 with Tom6 is most likely not unspecific. For Tom6, we found that PP2A and PP4 holoenzymes bind to Tom6 with and without Pfa. This could be explained by the holoenzymatic nature of both phosphatases. Especially among the PPP family, many of the catalytic subunits are associated with different regulatory subunits that regulate their activity, localization, and substrate specificity, making them particularly difficult to target and identify in the different holoenzymatic combinations [9, 47]. For these holoenzymes, the interaction is not only mediated through the catalytic subunit, and their activity needs to be regulated by means other than proximity alone. The additional interaction through regulatory subunits can increase the binding strength between phosphatase and substrate, and even override the intrinsic selectivity of the catalytic subunit [32, 48]. In humans, several short linear motifs (SLiMs) have been identified for the different PPPs that are necessary for the interaction between catalytic and regulatory subunits or that confer substrate specificity to different holoenzyme compositions (identified SLiMs for PPPs have recently been reviewed by [35, 49, 50]). Due to the conservation of several of these phosphatases between yeast and human, substrate selectivity has been shown for some and predicted for others to be regulated through the same motifs [28, 51, 52]. For both PP4 and PP2A, substrate specificity is regulated by association with regulatory subunits that interact with the substrates [10, 28, 32]. These binding sites of the substrates to the phosphatase regulatory subunits can be distal to the phosphorylation site [35]. In the case of Tom6, we show here that the binding sites are remarkably close to the phosphorylation sites. This demonstrates that trap‐peptides can be used if the interaction is not dependent on distant SLiMs. Our results show that PP2A and PP4 holoenzymes can associate with Tom6 regardless of the phosphorylation state of Ser16 and that the interaction is stable enough to enrich the phosphatases by affinity purification. This provides a new perspective on phosphatase substrate interactions, which are often viewed as weak and transient, and considered to be dependent on the phosphorylated residue [9, 53]. Another recent publication from our group showed that also with the substrate BAG3, phosphatases from the PPP family can be enriched by pulling on the non‐phosphorylated substrate [54]. However, follow‐up assays are required to confirm that the bound phosphatases dephosphorylate the substrate. For other phosphatase–substrate interactions, for example of multidomain‐serine/threonine‐specific phosphatases from other families [55], which rely on the presence of the phosphate group to bind the substrate or which are too weak to withstand the pulldown without it, the addition of Pfa may still be a solution to stabilize the interaction.

Although PP4 was highly enriched and Psy2^reg^ was shown to bind to Tom6, PP4/Psy2^reg^ did not dephosphorylate Tom6 *in vitro*. Thus, proximity to the phosphorylated site alone did not determine the activity of PP4. Potentially, either the intrinsic substrate specificity of the PP4 catalytic subunit or the conformation adapted upon binding to Tom6 could prevent the dephosphorylation. The binding of phosphatases through SLiMs to proteins can also lead to the dephosphorylation of other proteins localized nearby or in the same complex instead of the bound protein itself [32, 35, 56, 57, 58]. PP4 might target another TOM subunit for dephosphorylation that is in close proximity to Tom6 in the mature TOM complex (e.g., the FxxP motif is also present in Tom5 and Tom71 (SLiMSearch4 [59])).

Meanwhile, PP2A/Cdc55^reg^ was confirmed to dephosphorylate Tom6 by *in vitro* assays. In functional cellular studies, Harbauer *et al*. found that Tom6 Ser16 phosphorylation was cell cycle dependent, increasing during the M phase [2]. Godfrey *et al*. confirmed the temporal cell cycle‐dependent phosphorylation, noting a rapid increase following release from the G1 phase [6]. Therefore, Tom6 is expected to be dephosphorylated later in the cell cycle or during mitotic exit. In yeast, Cdc14 was thought to be the main phosphatase to regulate this process [60, 61]. Over the last decade, it has become increasingly clear that also PP2A also plays an important role in regulating the cell cycle at different stages. The different roles of PP2A during the cell cycle progression have been reviewed by Ariño *et al*. [62]. Several publications have identified potential PP2A/Cdc55^reg^ substrates by phospho‐proteomics of Cdc55^reg^ knock‐out yeast [6, 63, 64, 65, 66, 67]. However, Tom6 was only detected in one of the datasets with a slight increase in the knock‐out cell line compared to the wild type [67]. Additionally, Tom6 phosphorylation is cell cycle dependent which was not considered in the phosphoproteomic datasets of the Cdc55^reg^ knock‐out yeast. Since PP2A/Cdc55^reg^ is already known to be regulated in a cell cycle‐dependent manner, it seems likely that it dephosphorylates Ser16 of Tom6 at a specific cell cycle stage to regulate the assembly of the mature TOM complex.

To conclude, the identification of phosphatases that target specific phosphorylated residues in proteins remains a major challenge in signaling research. In this study, we used synthetic trap‐peptides containing Pfa or serine to identify phosphatases targeting pSer16 on Tom6. Both peptides significantly enriched several phosphatases, enabling the detection of PP2A and PP4 as full trimeric holoenzymes including their catalytic and two regulatory subunits. Thus, this approach clearly demonstrates that phosphatase–substrate interactions in these cases can be stable enough to withstand a pulldown and do not necessarily require the substrate to be phosphorylated at the site of interest for the interaction. The choice of the phosphomimetic can influence the interaction. In this study, the used Pfa even slightly reduced PP2A binding to the substrate. We found that the binding of the holoenzymes to Tom6 is mediated by the regulatory subunits Cdc55^reg^ and Psy2^reg^. While *in vitro* activity assays failed to demonstrate dephosphorylation of Tom6 by purified Psy2^reg^/Pph3^cat^ (PP4), purified Cdc55^reg^/Pph22^cat^ (PP2A) successfully dephosphorylated Tom6 phospho‐peptides and recombinantly expressed Tom6pSer^16^‐GST, showing that proximity is not the only determinant for dephosphorylation by PP4. Therefore, we propose that PP2A/Cdc55 acts as a negative regulator opposing Cdk1 phosphorylation at Ser16 of Tom6. Based on our results, synthetic trap‐peptides appear to be a promising tool to identify phosphatases targeting specific phosphorylation sites if the interaction is not dependent on distant SLiMs, which will aid in the identification of the reaction cycle of the kinase‐phosphatase interplay regulating a plethora of cellular signaling pathways.

## Materials and methods

### Solid phase peptide synthesis (SPPS)

Chemicals used were purchased from Carl Roth (Karlsruhe, Germany), Merck (Darmstadt, Germany), Sigma‐Aldrich (St. Louis, USA) and Biosynth (Staad, Switzerland) if not stated otherwise.

Peptides were synthesized on a 50 μmol scale. Rink Amide resin was swollen in N,N‐dimethylformamide (DMF) for 30 min before starting the coupling cycle of the first amino acid. The coupling of the first cysteine and the PEG linker was performed manually followed by automated peptide synthesis.

For the cysteine coupling, the beads were treated twice for 5 min with 2 mL 20% (v/v) piperidine in DMF on a shaker to remove the fluorenylmethyloxycarbonyl (Fmoc) protective group. The beads were thoroughly washed with DMF before adding Fmoc‐l‐cysteine (4 eq., 0.2 mmol), O‐benzotriazole‐*N*,*N*,*N′*,*N′‐tetramethyluronium*hexafluorophosphate (HBTU) (4 eq., 0.2 mmol), *N*‐hydroxybenzotriazole hydrate (HOBt) (4 eq, 0.2 mmol), and N‐methylmorpholine (NMM) (6 eq., 0.3 mmol) in 2 mL DMF. The coupling mix was shaken with the beads for 2 h. The beads were again washed with DMF and deprotected with piperidine as before. For the coupling of {2‐[2‐(Fmoc‐amino)ethoxy]ethoxy}acetic acid (Fmoc‐PEG_2_) (3 eq., 0.15 mmol), HOBt (3 eq., 0.15 mmol), HBTU (3 eq., 0.15 mmol), and NMM (6 eq., 0.3 mmol) were dissolved in 2 mL DMF and taken up in the syringe with the beads and coupled overnight.

The other amino acids were coupled using a MultiPep RSi peptide synthesizer (Intavis Bioanalytical Instruments, Cologne, Germany). Each coupling cycle begins with the deprotection of the previous amino acid with 20% (v/v) piperidine in DMF for 3 min followed by another incubation of 8 min. The coupling reaction was performed twice for 30 min for each Fmoc protected amino acid (4 eq., 0.2 mmol Fmoc‐amino acid, HOBt, HBTU and NMM). The termini to which the amino acid did not couple were capped through the addition of 5% (v/v) acetic anhydride (Ac_2_O) and 5% (v/v) 2,6‐lutidine in DMF for 5 min. As final steps, the last coupled amino acid was deprotected with piperidine and capped as described above. Between all steps, the beads were washed with DMF. Peptides were cleaved off the resin and fully deprotected in a single step by shaking overnight in cleavage cocktail (95.0% (v/v) trifluoroacetic acid (TFA), 2.5% (v/v) triisopropylsilane (TIPS), 2.5% (v/v) H_2_O). For the peptides for which this cleavage cocktail was not successful in removing all protective groups of the amino acid side chains, a cleavage cocktail containing ethane‐1,2‐dithiol (EDT) was used instead (94.0% (v/v) TFA, 2.5% (v/v) EDT, 2.5% (v/v) H_2_O, 1.0% (v/v) TIPS). The cleaved and deprotected peptides were precipitated in ice‐cold diethyl ether (Et_2_O, −20 °C) and pelleted by centrifugation (5 min, 4000 rpm, 4 °C). The remaining Et_2_O was left to evaporate under atmosphere.

The cleaved peptides were purified with a 1260 Infinity II Preparative (Prep) high‐performance liquid chromatography system (HPLC) (Agilent Technologies, Santa Clara, USA) with an acetonitrile (ACN) in H_2_O gradient (10 to 50 or 70% ACN) containing 0.05% (v/v) TFA. Validation of the peptide mass was done by Microflex LT matrix‐assisted laser desorption/ionization (MALDI) (Bruker, Billerica, USA) with an α‐cyano‐4‐hydroxycinnamic acid matrix and by 1260 Infinity I HPLC System with an EC 250/4 NUCLEODUR 100–5 C18 ec column (Macherey‐Nagel, Düren, Germany) with the same solvent composition as the Prep‐HPLC coupled to a 6120 Quadrupole LC/MS (Agilent Technologies, Santa Clara, USA) (HPLC‐MS). The purified peptide was dried using a lyophilizer VACO 2 (Zirbus, Bad Grund, Germany) and dissolved as a 10 or 5 mm stock solution in 10% (v/v) dimethyl sulfoxide (DMSO) in H_2_O. The peptide HPLC traces and MS spectra are shown in Figs S5–S15.

### Pfa deprotection and coupling

The protective groups were removed from (2 s)‐4‐(diethodxyphosphoryl)‐2‐({[(9 h‐fluoren‐9‐yl)methoxy]carbonyl}amino)‐4,4‐difluorobutanoic acid (Fmoc‐Pfa‐Et_2_) (Enamine, Frankfurt am Main, Germany). Fmoc‐Pfa‐Et_2_ (4 eq., 0.2 mmol) was dissolved in 2 mL dichloromethane (DCM), and 0.5 mL Bromo‐trimethylsilane (BrTMS) was added. The reaction was stirred for 4 h. The solvent was removed by rotary evaporator, and the reaction was repeated overnight with fresh DCM and BrTMS. After the second deprotection step, the dry product was dissolved in 2–3 mL H_2_O followed by lyophilization to obtain Fmoc‐Pfa.

The resin‐coupled peptide was washed several times with DCM and dried under vacuum for several hours. To increase coupling efficiency, the reaction was performed under argon atmosphere. The dried Fmoc‐Pfa, HOBt (4 eq., 0.2 mmol), HBTU (4 eq., 0.2 mmol) were dissolved in 2 mL dry DMF. N,N‐Diisopropylethylamine (DIPEA) (12 eq., 0.6 mmol) was added; the coupling mix was taken up in the reactor syringe with the peptide and placed on a shaker overnight. The resin was washed several times with DMF.

### Yeast cytosolic extracts

YPH499 yeast was grown in YPG medium (1% (w/v) yeast extract, 2% (w/v) bacto peptone, 3% (w/v) glycerol) overnight at 30 °C and 130 rpm until an OD_600_ of 1–2.5 was reached. Yeast cells were pelleted (10 min, 4000 rpm), washed with deionized water, and pelleted again (5 min, 4000 rpm). The pellet was resuspended in 2 mL/g of cells in dithiothreitol (DTT) buffer (10 mm DTT, 100 mm Tris‐H_2_SO_4_ pH 9.4) and incubated for 20 min at 30 °C and 130 rpm. The cells were washed with 1.2 m sorbitol (5 min, 4000 rpm) before being resuspended in zymolyase buffer (1.2 m sorbitol, 20 mm KP_i_, pH 7.4) with zymolyase (3–4 mg per g of cells). Zymolyase (Nacalai tesque, Kyoto, Japan) treatment was performed for 30–60 min at 30 °C and 130 rpm. Cells were harvested by centrifugation (5 min, 4000 rpm) and washed again with 1.2 m sorbitol. From here on, all steps were performed on ice and in centrifuges precooled to 4 °C. For lysis, the cells were resuspended in homogenizing buffer (10 mm Tris/HCl pH 7.4, 0.6 m sorbitol, 1 mm phenylmethylsulfonyl fluoride (PMSF)) and transferred to a precooled potter. Cells were lysed by pottering 25 times up and down. Lysates were centrifuged for 2 min at 1500 rpm, directly followed by 4 min at 3000 rpm. The supernatant was transferred to a new reaction tube and centrifuged for 5 min at 4000 rpm. For the final centrifugation step, the supernatant was transferred to a fresh reaction tube and centrifuged for 15 min at 12 000 rpm. The supernatant was snap frozen in liquid nitrogen or immediately used.

### SulfoLink pull‐down with yeast cytosolic extracts

SulfoLink coupling resin (Thermo Fisher Scientific, Waltham, USA) and coupling buffer (50 mm Tris/HCl pH 8.5, 5 mm EDTA) were pre‐warmed to room temperature (RT). SulfoLink bead slurry was centrifuged at 20 000 *g*, and the supernatant removed. The beads were washed three times with coupling buffer. Peptides were dissolved to a final concentration of 0.29 mm in coupling buffer. For 200 μL of initially added bead slurry, 1 mL of dissolved peptide was added. For the negative control, only coupling buffer was added, and the beads were incubated overnight at RT on a rotating wheel. Coupling efficiency of the peptide was determined by BCA assay (Pierce, Thermo Fisher Scientific, Waltham, USA) comparing the dissolved peptide solution before and after incubation with the beads. Beads were washed twice with coupling buffer. 50 mm l‐cysteine dissolved in coupling buffer was added to the beads and incubated for 1 h at RT while rotating. Again, the beads were washed twice with coupling buffer.

Before adding the cytosolic fractions, the beads were washed twice with homogenizing buffer. From here on, all steps were performed on ice and the centrifugation steps at 4 °C. 1 mL of the cytosolic fraction (for the assay to be analyzed by MS the lysate concentration was adjusted to 0.5 mg·mL^−1^ and for the assay analyzed by western blot to 2 mg·mL^−1^) was added to the beads and rotated for 1 h. To remove unspecifically bound proteins, the beads were centrifuged for 2 min at 3000 rpm. The supernatant was removed and carefully 1 mL wash buffer (150 mm NaCl, 10 mm Tris/HCl pH 7.5) was added and inverted once. This washing step was repeated twice more. After the last step, 100 μL 2× reducing sample buffer (125 mm Tris, 20% (v/v) glycerol, 4% (w/v) SDS, 0.02% (w/v) bromphenol blue, 5% (v/v) β‐mercaptoethanol) was added to the beads and boiled for 10 min at 95 °C. The assay was performed in triplicates and analyzed by mass spectrometry (MS).

### SDS‐gel and western blot

Nu‐Page 4–12% protein gels (Invitrogen, Waltham, USA) were run in MOPS buffer (0.05 m MOPS, 0.05 m Tris, 3.5 mm SDS, 1.0 mm EDTA) at 150–200 V.

To stain proteins in the gel, it was incubated in colloidal Coomassie stain overnight [68] and destained with H_2_O for several hours.

Gels with ^35^S‐labeled samples were stained with colloidal Coomassie before being dried onto Whatman paper. Once dried, the gels were exposed with PhosphorImager screens (GE Healthcare, Düsseldorf, Germany) for 24 h to 4 days. The screens were imaged by digital autoradiography (PhosphorImager, GE Healthcare, Düsseldorf, Germany).

For western blots, the proteins were transferred onto a polyvinylidene difluoride (PVDF) membrane (Merck Millipore, Burlington, USA) by wet transfer (25 mm Tris, 0.2 m glycine, 20% methanol (v/v)) for 1 h at 100 V. Membranes were blocked for 1 h at RT with Tris‐buffered saline containing 0.1% Tween‐20 (TBS‐T) and 5% (w/v) non‐fat milk powder. After the blocking, the membranes were washed with TBS‐T before adding the primary antibody overnight at 4 °C. All antibodies used in this work are listed in Table 1. Blots were washed three times for 10 min with TBS‐T and incubated with the secondary antibody HRP‐conjugated anti‐rabbit or mouse IgG for 1 h at room temperature. The membranes were washed with TBS‐T (three times 10 min) before being developed with Western Lightning Plus‐ECL Enhanced Chemiluminescence Substrate (Perkin Elmer, Waltham, USA). Imaging of membranes and gels was done with a Fusion FX Imaging System (Vilber, Eberhardzell, Germany).

**Table 1:** Antibodies used in this study.

Primary/secondary	Target protein	Species	Stock solution	Origin
Primary	pTom6 Ser16^a^	Rabbit	1:100 in TBS‐T	Eurogentec (Seraing, Belgium) EG11139 [2]
	GST	Mouse	1:1000 in TBS‐T+ 5% BSA	ThermoFisher (Waltham, USA) MA4‐004
	Cdc55	Mouse	1:100 in TBS‐T+ 5% BSA	Prof. Egon Ogris Center for Medical Biochemistry, Max Perutz Labs, Vienna Clone 9D3‐H6 [69]
	Pph21	Rabbit	1:10 000 in TBS‐T+ 5% BSA	Prof. Egon Ogris Center for Medical Biochemistry, Max Perutz Labs, Vienna [70]
	Tpd3	Mouse	1:100 in TBS‐T+ 5% BSA	Prof. Egon Ogris Center for Medical Biochemistry, Max Perutz Labs, Vienna Clone 5G2 [25]
Secondary, HRP coupled	ProtA	Rabbit	1:200 in TBS + 5% milk	Sigma‐Aldrich (St. Louis, USA) P1291
	Mouse IgG	Goat	1:10000 in TBS‐T+ 5% non‐fat milk	Sigma‐Aldrich (St. Louis, USA) A0168
	Rabbit IgG	Donkey	1:10000 in TBS‐T+ 5% non‐fat milk	Sigma‐Aldrich (St. Louis, USA) GENA934‐1ML

^a^ The antibody was double affinity‐purified from serum by the company to ensure specificity.

Blots were quantified using fiji/imagej (version 2.12.0) and the statistical analysis was performed with GraphPad Prism 6.

### Mass spectrometry (MS)‐based proteomics

These methods have been described similarly in previous publications [71, 72].

Sample preparation: In‐gel trypsin digestion was performed according to standard procedures [73]. Briefly, the SulfoLink pull‐downs as well as the purified PP4 and PP2A holoenzymes were prepared for SDS‐gel analysis as described above, and then run on a Nu‐PAGE™ 4%–12% Bis‐Tris protein gel (ThermoFisher Scientific, Waltham, USA) for only about 1 cm. Subsequently, the still not size‐separated single protein band per sample was cut out, reduced (50 mm dithiothreitol), alkylated (55 mm chloroacetamide) and digested overnight with trypsin (Trypsin Gold, mass spectrometry grade, Promega, Madison, WI, USA). The peptides obtained were dried to completeness and resuspended in 25 μL of 2% acetonitrile, 0.1% formic acid in HPLC grade water. Finally, 5 μL of sample were injected per mass spectrometric (MS) measurement.

Mass spectrometric measurements: LC–MS/MS data acquisition of the SulfoLink pull‐downs was carried out on a Dionex Ultimate 3000 RSLCnano system coupled to a Q‐Exactive HF‐X mass spectrometer (ThermoFisher Scientific, Bremen, Germany). Injected peptides were delivered to a trap column (ReproSil‐pur C18‐AQ, 5 μm, Dr. Maisch, 20 mm × 75 μm, self‐packed) at a flow rate of 5 μL/min in 0.1% formic acid in HPLC grade water. After 10 min of loading, peptides were transferred to an analytical column (ReproSil Gold C18‐AQ, 3 μm, Dr. Maisch, 450 mm × 75 μm, self‐packed) and separated using a 50 min gradient from 4% to 32% of solvent B (0.1% FA, 5% DMSO in acetonitrile) in solvent A (0.1% FA, 5% DMSO in HPLC grade water) at a flow rate of 300 nL/min The Q‐Exactive HF‐X mass spectrometer was operated in data‐dependent acquisition (DDA) and positive ionization mode. MS1 spectra (360–1300 m/z) were recorded at a resolution of 60 k using an automatic gain control (AGC) target value of 3e6 and maximum injection time (maxIT) of 45 msec. Up to 18 peptide precursors were selected for fragmentation. Only precursors with charge states 2 to 6 were selected, and dynamic exclusion of 30 s was enabled. Peptide fragmentation was performed using higher energy collision‐induced dissociation (HCD) and a normalized collision energy (NCE) of 26%. The precursor isolation window width was set to 1.3 m/z. MS2 resolution was 15,000 with an automatic gain control (AGC) target value of 1e5 and maximum injection time (maxIT) of 25 msec. LC–MS/MS data acquisition of the purified PP4 and PP2A holoenzymes was carried out on a Dionex Ultimate 3000 RSLCnano system coupled to an Orbitrap Fusion LUMOS mass spectrometer (ThermoFisher Scientific, Bremen, Germany). LC and MS parameters on this instrument resembled the ones described above, except: MS1 normalized automatic gain control (AGC) target value of 100%, maximum injection time (maxIT) of 50 msec, fixed cycle time of 2 s, normalized collision energy (NCE) of 30%, MS2 normalized automatic gain control (AGC) target value of 150%, and maximum injection time (maxIT) of 22 msec.

Mass spectrometric data analysis: Peptide identification and quantification was performed using the software MaxQuant (version 1.6.3.4 [74]) with its built‐in search engine Andromeda [75]. MS2 spectra were searched against the Uniprot *Saccharomyces cerevisiae* protein database (UP000000589, 6049 protein entries, downloaded November 2020), supplemented with common contaminants (built‐in option in MaxQuant). Trypsin/P was specified as the proteolytic enzyme. Carbamidomethylated cysteine was set as a fixed modification. Oxidation of methionine and acetylation at the protein N terminus was specified as variable modifications. Results were adjusted to 1% false discovery rate on peptide spectrum match (PSM) level and protein level employing a target‐decoy approach using reversed protein sequences. Label‐Free Quantification (LFQ [76]) intensities were used for protein quantification with at least 2 peptides per protein identified. The minimal peptide length was defined as 7 amino acids, and the “match‐between‐runs” functionality was disabled. The MaxQuant output data were analyzed with Perseus (version 1.6.15.0). The LFQ values were filtered to remove proteins only identified by site, reverse, and potential contaminants. The remaining values were log_2_ transformed. Rows were filtered by valid values (3 values in at least one group). The missing values were imputed from a normal distribution (width 0.3, down shift 1.8). The results were visualized in volcano plots with a false discovery rate (FDR) of 5% and a fold change of 0.1. Volcano plots were generated with ggplot2 and tidyverse in R (version4.3.3). For the MS data of the phosphatases purified through Psy2^reg^ and Cdc55^reg^, the LFQ values were filtered as before. The next analysis steps were not performed as they are individual samples without biological triplicates.

### Isolation of yeast genomic DNA

Yeast cells were grown in 5 mL YPD medium (1% (w/v) yeast extract, 2% (w/v) bacto peptone, 2% (w/v) glucose) to reach an OD_600_ of 0.5–0.7. Cells were harvested by centrifugation (5 min, 4000 rpm), resuspended in 150 μL solution A (50 mm Tris/HCl pH 7.5, 10 mm EDTA, 0.3% β‐mercaptoethanol, 0.50–0.25 mg·mL^−1^ zymolyase (Nacalai tesque)). And incubated at 37 °C for 1 h. Then, 20 μL 10% SDS were added and carefully vortexed. 100 μL 8 m NH_4_ acetate were added and the samples incubated at −20 °C for 15 min. Cell debris were removed by centrifugation (15 min, 14 000 ***g***, 4 °C). The supernatant was transferred to a fresh reaction tube; 20 μL isopropanol were added and centrifuged as before. The pellet was washed with 70% ethanol and left to dry before being resuspended in 20–30 μL H_2_O.

### Expression of ^35^S methionine‐labeled phosphatase subunits

For the expression of the individual subunits of the phosphatases PP4 (Psy2^reg^, Psy4^reg^, Pph3^cat^) and PP2A (Pph21^cat^, PPh22^cat^, Cdc55^reg^, Tpd3^scaf^) the genes were amplified from yeast genomic DNA. Forward primers contain an overhang with an SP6 promoter site and the rabbit Kozak sequence before the gene‐specific sequence before the start codon (all primers used in this work are listed below Table 2). The reverse primers bind just after the stop codon of the gene of interest. The amplifying PCR was carried out with Phusion Polymerase (Thermo Fisher, Waltham, USA), yeast genomic DNA, an annealing temperature of 59 °C, and an elongation time of 3 min. PCR product sizes were confirmed in a 1% agarose gel. PCR products were purified with the NucleoSpin PCR Clean‐up kit (Machery‐Nagel) and eluted with RNase‐free H_2_O. The transcription of the PCR product to RNA was performed according to the manufacturer's instructions (mMESSAGE mMACHINE kit, Thermo Fisher, Waltham, USA). For the purification of the RNA products, the RNA‐MEGAclear kit (Thermo Fisher, Waltham, USA) was used.

**Table 2:** Primer sequences. All primers were ordered from Eurofins (Luxembourg, Luxembourg) or Mycrosynth (Balgach, Switzerland).

Name	Sequence 5′‐3′
Psy2 Fwd	TCGATTTAGGTGACACTATAGAATACGCCGCCGCCATGTCATTACCGGGTACACC
Psy2 Rev	CTTCTTTCCAACAAGGAAAAACTCAAG
Psy4 Fwd	TCGATTTAGGTGACACTATAGAATACGCCGCCGCCATGAGCTCGACGATGTTGG
Psy4 Rev	GTAGAGAAGTCATCTCTCGATCATC
Pph3 Fwd	TCGATTTAGGTGACACTATAGAATACGCCGCCGCCATGATGGACTTAGATAAG
Pph3 Rev	GACAATTAGAGTGCCTGTTAAAAATTTATAAG
Pph21 Fwd	TCGATTTAGGTGACACTATAGAATACGCCGCCGCCATGGATACAGATTTAGATGTGCC
Pph21 Rev	CTATATAGATGCATATATGTATACATACTC
Pph22 Fwd	TCGATTTAGGTGACACTATAGAATACGCCGCCGCCATGGATATGGAAATTGATGACCC
Pph22 Rev	GTAAGGATAAAGGTGTAATAGATATATATTATAAG
Cdc55 Fwd	TCGATTTAGGTGACACTATAGAATACGCCGCCGCCATGGCACAAAACAATTTTG
Cdc55 Rev	GGGAAAATAAGGAATTATTATAATTATAATGCGG
Psy2 ProtA Fwd	TCTGGTGATC AGTTAGCATT TAAAAAAAGC GTTGACCAAA TGAATGCAAG TACT CGT ACG CTG CAG GTC GAC
Psy2 ProtA Rev	ACAACCATGACCGTTGTGCTAGCTTTTTATTCTTCTTTCCAACAAGGAAAAAC ATC GAT GAA TTC GAG CTC G
Cdc55 ProtA Fwd	GAAAATAGTATTGCTGTTGCAGCAACTAATAATTTATTCATTTTTTCCGCATTA CGT ACG CTG CAG GTC GAC
Cdc55 Port A Rev	GTGGGGAAGATATGGGATAAAAAAAAGTAAGGGAAAATAAGGAATTATTATAA ATC GAT GAA TTC GAG CTC G
Tom6 Ser16 → TAG Fwd	TTTGGTTGTTGTGGCTAGGCCGCACCTGCAGC
Tom6 Ser16 → TAG Rev	GCTGCAGGTGCGGCCTAGCCACAACAACCAAA

These mRNAs were used with the Flexi Rabbit Reticulocyte Lysate System (Promega) to express the proteins with ^35^S methionine. Expected protein sizes of the expressed proteins are: Psy2 98.0 kDa, Psy4 50.6 kDa, Pph3 35.2 kDa, Pph21 41.9 kDa, Pph22 42.0 kDa, Cdc55 59.7 kDa, and Tpd3 70.9 kDa. The expression of Tpd3 was not successful.

### SulfoLink pull‐down with individual phosphatase subunits

Peptides were coupled to the SulfoLink beads as described before. 40 μL bead slurry was used for each pull‐down. Instead of adding the cytosolic fractions, the expressed subunits were added to the beads diluted in homogenizing buffer (1–3 μL depending on the expression efficiency). All incubation and washing steps were performed as for the pull‐down with yeast cytosolic extracts.

For the statistical analysis of the quantified blots, a one‐way ANOVA followed by a Tukey's multiple comparisons test was performed.

### Tagging of Psy2^reg^ and Cdc55^reg^ with ProtA

Psy2^reg^ and Cdc55^reg^ were tagged with ProtA according to the protocol described by Knop *et al*. and Janke *et al*. [77, 78]. The inclusion of the tag was selected with a nourseothricin resistance for Psy2^reg^ using a pFA6a TEV‐ProtA‐Nat plasmid and a kanamycin resistance for Cdc55^reg^ with pFA6a TEV‐ProA‐KanMX6 [75, 76]. PCR amplification of the tag with the overhangs binding to the protein of interest was performed with Phusion Polymerase, an annealing temperature of 55 °C, and an elongation time of 3 min. Used primers are listed in Table 2. The PCR products were purified using the NucleoSpin PCR Clean‐up kit.

Psy2^reg^ was tagged in BY4741 wild‐type yeast and in BY4741 *pph3*Δ (Euroscarf, Oberursel, Germany), while Cdc55^reg^ was only tagged in the wild‐type yeast. To transform the yeast cells with the PCR products, they were grown overnight in YPD medium (30 °C, 130 rpm). The cultures were diluted in the morning in 50 mL to an OD_600_ of 0.4 and grown until reaching a final OD_600_ of 0.8–1.0. Cells were harvested by centrifugation (5 min, 4000 rpm) and washed once with sterile H_2_O. The pellet was resuspended in 500 μL sterile 0.1 m lithium acetate (LiAc). 100 μL of this suspension were aliquoted for each transformation. 10 μL of carrier DNA (Herring sperm DNA, Promega) as well as 2 μg of the PCR product were added. To each sample, 1 mL LiAc‐PEG400 (2.5% PEG400, 100 mm LiAc) was added and the samples were incubated for 30 min at 30 °C and 350 rpm. To increase transformation efficiency, 37 μL DMSO were added. Heat shock was performed by placing the cells for 25 min at 42 °C. Cells were pelleted (5 min, 4000 rpm), resuspended in 5 mL YPD, and grown for 2–3 h (30 °C, 130 rpm). Cells were again pelleted and streaked onto a YPD plate with the corresponding antibiotic. The plates were placed at 30 °C until colonies appeared. Individual colonies were subjected to a second round of selection on fresh plates. The incorporation of the tag was confirmed by western blot with a primary antibody against ProtA.

### Purification of Psy2^reg^ProtA

**Fig. 5:**
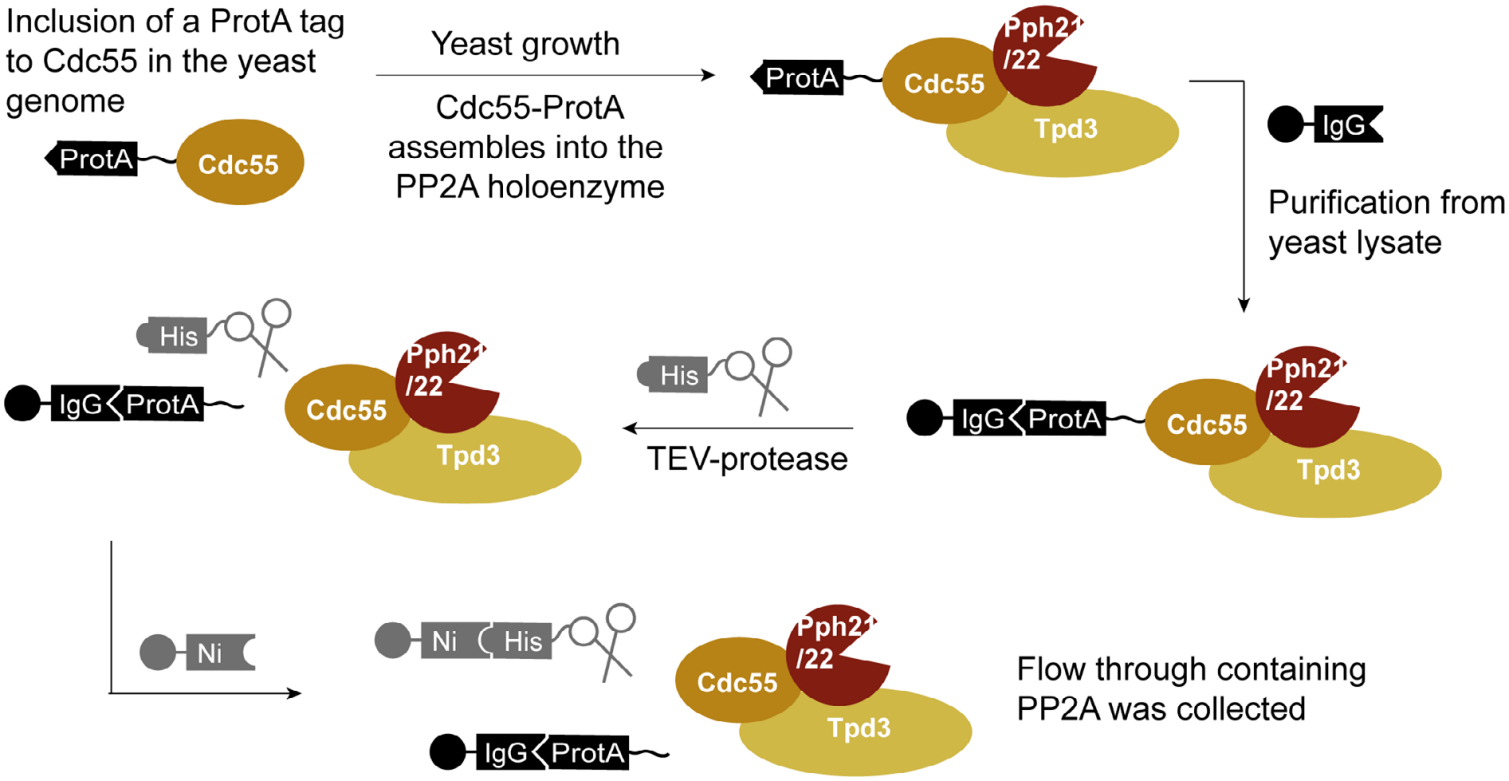
Purification workflow of Cdc55^reg^/Pph22^cat^ PP2A through Cdc55^reg^‐ProtA. The procedure is the same for the Psy2^reg^/Pph3^cat^ purification through Psy2^reg^‐ProtA instead of Cdc55^reg^‐ProtA. For PP4, the other subunits are Pph3 instead of Pph21/22 and Psy4 instead of Tpd3.

The isolation protocol was adapted from Ma *et al*. [29] (Fig. 5). Starter cultures of the yeast strains with the tagged Psy2 (BY4741 wt and *pph*3Δ) were diluted in 3.2 L of YPD medium to reach an OD_600_ of 2 the next day when grown at 30 °C. Yeast cells were harvested by centrifugation (10 min, 4000 rpm) and washed once with H_2_O.

From here on, all steps were performed on ice and centrifugation at 4 °C. The pellets were resuspended in 10 mL lysis buffer (20 mm) Tris/HCl pH 7.5, 10% (v/v) glycerol, 200 mm potassium acetate, 1 mm EDTA, 1× protease inhibitor cocktail (EDTA free) (Sigma‐Aldrich, St. Louis, USA). The suspension was frozen dropwise in liquid nitrogen. Frozen cells were ground for 10 min at 25 Hz by cryomill (Retsch, Haan, Germany). The powder was thawed on ice, and a clarifying centrifugation step was performed (10 min, 15 000 ***g***).

Meanwhile, 400 μL IgG beads were washed twice with 500 μL acetate buffer pH 3.5 in Mobicol columns (Biozol, Hamburg, Germany) followed by two washing steps with lysis buffer. Washed beads were mixed with yeast lysate and rotated for 2–4 h at 4 °C. The beads were then washed five times with lysis buffer and once with TEV reaction buffer (10 mm Tris/HCl pH 8.0, 150 mm NaCl, 0.1% (v/v) Igepal, 0.5 mm EDTA, 1 mm DTT). 1.5 mL TEV reaction buffer and 100 μL TEV protease (1 mg·mL^−1^, EMBL Protein Expression and Purification Core Facility) were added to the beads and incubated overnight at 4 °C.

200 μL Ni‐NTA bead suspension (Thermo Fisher Scientific, Waltham, USA) was washed once with H_2_O followed by two washing steps with TEV reaction buffer. These beads were then added to the IgG beads with the TEV protease and rotated for 1 h. The flow through containing the product was dialyzed to storage buffer (2 mm Tris/HCl pH 7.5, 100 mm NaCl, 5 mm MnCl_2_, 0.5 mm DTT). The samples were then supplemented with 10% (v/v) glycerol, aliquoted, and snap frozen in liquid nitrogen. The products had a final concentration measured at the nanodrop of 0.546 mg·mL^−1^ for Psy2^reg^/Pph3^cat^ and 0.469 for Psy2^reg^ *pph*3Δ. The full trimeric holoenzyme has a molecular mass of 184.1 kDa.

### Purification of Cdc55^reg^‐ProtA

The isolation protocol was adapted from Sarkar *et al*. [30] (Fig. 5). BY4741 wt and Cdc55^reg^‐ProtA were grown in 5 mL YPD at 30 °C, 130 rpm overnight. Starter cultures were diluted in 3.2 L of YPD medium to reach an OD_600_ of 1–2 in the morning and grown overnight at 30 °C, 130 rpm. Cells were harvested by centrifugation (10 min, 4000 rpm) and washed with H_2_O. The pellet was resuspended in 20 mL lysis buffer (50 mm Tris/HCl pH 7.5, 100 mm NaCl, 1 mm EDTA, 1% (v/v) Igepal, 1 mm PMSF, 1× protease inhibitor cocktail (EDTA free)). For lysis, 6 g of glass beads (0.5 mm diameter, Roth, Karlsruhe, Germany) per g of cells were added and the cells were broken by bead beating (4 m/s for 20 s, FastPrep‐24 5G homogenizer (MP Biomedicals, Santa Ana, USA)). Beads were removed by centrifugation (150 g, 5 min) followed by a centrifugation to clarify the lysate (16 000 rpm, 6 min).

0.4 mL of IgG bead suspension was prepared as described for the purification of Psy2^reg^‐ProtA. The cleared lysate was incubated with the beads for 2 to 4 h at 4 °C while rotating. The cleavage of the product by TEV protease was performed as described previously for Psy2^reg^‐ProtA. The buffer of the product was exchanged to storage buffer (10 mm NaCl, 50 mm Tris/HCl pH 7 5, 1.5 mm MgCl_2_, 1 mm DTT) by dialysis. The product was supplemented with 10% (v/v) glycerol, aliquoted, and snap frozen in liquid nitrogen. The products had a final concentration measured at the nanodrop of 0.791 mg·mL^−1^ for no ProtA tag and 0.814 mg·mL^−1^ for Cdc55^reg^/Pph22^cat^. The full trimeric holoenzyme has a molecular mass of 173.6 kDa. If the product only contained fully assembled PP2A holoenzymes, the stocks would have a concentration of 4.69 μm.

### Malachite green assay

In 96‐well plates, reaction mixtures were prepared in a final volume of 50 μL. 7.5 nmol of the phospho‐peptide was added in the corresponding reaction buffer to reach a final concentration of 150 μm (reaction buffer for PP2A: 100 mm NaCl, 50 mm Tris/HCl pH 7.5, 1.5 mm MgCl_2_; 1 mm DTT; for PP4: 25 mm Tris/HCl pH 7.5, 100 mm NaCl, 5 mm MnCl_2_, 0.5 mm DTT). Of the purified Psy2^reg^/Pph3^cat^ 10 μL, Cdc55^reg^/Pph22^cat^ 5 μL and of the fast alkaline phosphatase (FastAP, Thermo Fisher Scientific, Waltham, USA) 1 μL were added per well. Controls with only the phosphatase, the buffer, or the peptides were included. Samples were prepared in triplicates per plate for each replicate. Samples were incubated at 30 °C for 1 h before adding 100 μL of Biomol green reagent (Enzo, Lörrach, Germany). For the malachite green to react with the free phosphate, the samples were incubated for 20 min at RT. The absorbance at 620 nm was measured by Synergy H1 microplate reader (BioTek, Bad Friedrichshall, Germany). Each condition was added in triplicates on the 96‐well plate, and the assay was performed in triplicates. Of the obtained values, the blank without peptide was deducted. For the statistical analysis, a one‐way ANOVA followed by a Tukey's multiple comparisons test was performed in GraphPad Prism 6.

For the competition assay between Tom6^pS16^ and Tom6^PfaNT^ and Cdc55^reg^/Pph22^cat^, the phosphatase was preincubated with 0, 150, 500, or 1000 μm Tom6^PfaNT^ for 10 min at room temperature before adding Tom6^pS16^.

### DiFMUP (6,8‐Difluoro‐4‐Methylumbelliferyl phosphate) synthesis

**Fig. 6:**
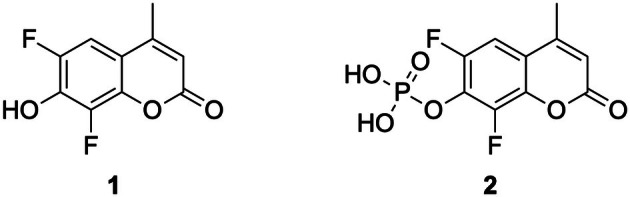
Structures of DiFMU (1) and DiFMUP (2).

**1** (Fig. 6) was synthesized following the protocol published by [79].

**1**: 2,4‐Difluorobenzene‐1,3‐diol (100 mg, 0.684 mmol, 1 eq.) (Abcr, Karlsruhe, Germany) and Ethyl acetoacetate (86.5 μL, 0.684 mmol, 1 eq.) were charged in a flask and cooled to 0 °C. Methanesulfonic acid (1.11 mL, 17.11 mmol, 25 eq.) was added dropwise over 5 min. After 30 min, the reaction was allowed to come to rt. After 3 h at rt, the reaction was cooled to 0 °C and quenched with 20 mL H_2_O. A precipitate formed, which was filtered off and washed with water. The white solid was dried under high vacuum and used for the next reaction without further purification. Yield: 118 mg, 81.3%, 0.556 mmol. ^**1**^ **H NMR** (400 MHz, CDCl_3_, δ): 7.14 (dd, *J* = 2.1, 10.30 Hz, 1H), 6.26 (s, 1H), 2.39 (s, 3H).

**2** (Fig. 6): 1 (118 mg, 0.556 mmol) was charged in a flask under Ar‐atmosphere, dissolved in 2 mL Pyridine, and cooled to 0 °C. Phosphorus(V) oxychloride (78 μL, 0.834 mmol, 1.5 eq.) was added as one portion. After 20 min at 0 °C, the reaction was allowed to come to rt. After 2 h at rt, it was cooled to 0 °C and quenched with 5 mL H_2_O. The reaction was filtered through a syringe filter (0.2 μm PTFE membrane, VWR) and directly injected into preparative HPLC for purification. For the HPLC‐Prep conditions, see section Solid phase peptide synthesis. A 10 to 70% ACN gradient was used for separation. Product fractions were collected and freeze‐dried. The solid was dissolved in 5 mL HCl (1 m) and purified via preparative HPLC again. Yield: 73 mg, 44.9%, 0.25 mmol, white powder. Measured m/z by HPLC‐MS 293.0 [M + H]^+^ (calculated m/z 293.0 [M + H]^+^). C_10_H_7_F_2_O_6_P. ^**1**^ **H NMR** (400 MHz, DMSO, δ): 7.65 (dd, *J* = 2.0, 10.7 Hz, 1H), 6.51 (s, 1H), 2.41 (d, *J* = 1.2 Hz, 3H). ^**31**^ **P NMR** (160 MHz, CDCl3, δ): −5.71 (s). ^**19**^ **F NMR** (375 MHz, CDCl3, δ): −130.4, −145.6.

NMR spectra were recorded with a Bruker Avance NeoAVANCE NEO 400 MHz NMR spectrometer with autosampler (^1^H: 400 MHz; ^31^P: 160 MHz; ^19^F: 374 MHz).

The NMR and MS spectra of the synthesized DiFMUP are shown in Figs S16–S20.

### DiFMUP assay

The effect of Tom6^Wt^ and Tom6^Pfa^ on PP2A activity was tested with DiFMUP (synthesized as described above) as substrate. In a black 96‐well plate, 5 μL of purified Cdc55^reg^/Pph22^cat^ were incubated in reaction buffer (100 mm NaCl; 50 mm Tris/HCl pH 7.5; 1.5 mm MgCl_2_; 1 mm DTT) together with 150 or 500 μM Tom6^wt^ or Tom6^Pfa^ for 10 min at room temperature. Then, 100 nm DiFMUP was added, reaching a final volume of 100 μL. Immediately after adding DiFMUP, the plate was placed in the plate reader preheated to 30 °C, and the fluorescence was measured every 30 s (excitation 358 nm, emission 452 nm, gain 60). Each condition was added in duplicates on the plate. From the measured values, the ones from the negative control with only DiFMUP were subtracted. The linear slope was calculated for each replicate. For the statistical analysis, a one‐way ANOVA followed by a Tukey's multiple comparisons test was performed, comparing all conditions to the control without peptide.

### Expression and purification of Tom6pSer^16^ ‐GST in *E. coli*

Plasmids for the expression of the cytosolic domain of Tom6 with a GST tag generated by Harbauer *et al*. were used [2]. For the introduction of a pSer at position 16 in the Tom6 sequence, an amber stop codon was introduced with the QuikChange lightning kit (Agilent, Santa Clara, USA) according to the manufacturer's protocol (sequences or the primers are listed above in Table 2).

pETGEX Tom6cyt(TAG)‐GST plasmids were transformed in competent BL21(DE3) *E. coli* together with SepRS/ tRNA^Sep^_CUA_ pair for the incorporation of pSer at the amber stop codon (plasmids for the expression of SepRS/tRNA^Sep^_CUA_ were kindly provided by Prof. Dr. Andreas Marx, University of Konstanz [22]). *E. coli* containing all three plasmids were selected on LB plates containing ampicillin, kanamycin, and chloramphenicol. A starter culture of one clone was grown in LB medium containing the three antibiotics at 37 °C and 180 rpm overnight. The preculture was diluted 1:50 in 2 L pre‐warmed LB medium and grown at 37 °C, 180 rpm until an OD_600_ of 0.6 was reached. To induce the expression, the medium was supplemented with 0.04% arabinose and 1 mm IPTG. For the introduction of pSer, 2 mm O‐Phospho‐l‐serine (Sigma‐Aldrich, St. Louis, USA) was added at the time point of the induction and another 2 mm 30 min later. The cultures were grown for 5 to 6 h at 37 °C and 180 rpm. Cells were harvested by centrifugation (30 min, 6500 ***g***).

The pellet was resuspended in lysis buffer (20 mm Tris/HCl pH 7.9, 30 mm NaCl, 10 mm β‐mercaptoethanol, 2 mm MgCl_2_, 10 mm imidazole, 0.5 mg·mL^−1^ lysozyme, 10 μg·mL^−1^ DNaseI, 1× protease inhibitor cocktail (EDTA free)). The bacteria were lysed by sonication (two times 2 min at 60% intensity) on ice. Lysates were cleared by centrifugation (45 min, 16 000 ***g***). 2 mL Ni‐NTA bead suspension (Invitrogen, Carlsbad, USA) were washed with H_2_O followed by binding buffer (20 mm Tris/HCl pH 7.9, 300 mm NaCl, 1 mm β‐mercaptoethanol, 10 mm imidazole). The cleared lysate was incubated with the beads for 1 h at 4 °C on a rocker. The flow through was collected, and the beads were washed three times with binding buffer. Bound protein was eluted in two steps, first with 5 mL elution buffer A (binding buffer with 200 mm imidazole, the pH was adjusted to 7.9) followed by 5 mL elution buffer B (binding buffer with 500 mm imidazole, the pH was adjusted to 7.9). The fractions containing the product were pooled and dialyzed against storage buffer (300 mm NaCl, 20 mm Tris/HCl, pH 7.2). The protein was diluted to a final concentration of 1 mg·mL^−1^, supplemented with 10% (v/v) glycerol, aliquoted, and frozen at −80 °C.

### Dephosphorylation of recombinant Tom6

To test the ability of phosphatases to dephosphorylate the recombinantly expressed Tom6pSer^16^‐GST, 1 μg of Tom6pSer^16^‐GST was incubated in the corresponding reaction buffer (PP2A: 100 mm NaCl, 50 mm Tris/HCl pH 7.5, 1.5 mm MgCl_2_; 1 mm DTT; PP4: 25 mm Tris/HCl pH 7.5, 100 mm NaCl, 5 mm MnCl_2_, 0.5 mm DTT) and the phosphatases (Psy2^reg^/Pph3^cat^: 10 or 19 μL, Cdc55^reg^/Pph22^cat^: 10 μL, Fast AP: 1 μL) in a total volume of 20 μL. Samples were incubated for the indicated time points at 30 °C and 350 rpm. They were mixed with 2× reducing sample buffer (125 mm Tris, 20% (v/v) glycerol, 4% (w/v) SDS, 0.02% (w/v) bromphenol blue, 5% (v/v) β‐mercaptoethanol) and boiled for 5 to 10 min at 95 °C. The phosphorylation levels were determined with antibodies binding pTom6 Ser16 and GST.

## Conflict of interest

The authors declare no conflict of interest.

## Author contributions

MK and CM conceived and supervised the study; LS, MK, CM, and NV designed experiments; LS performed experiments and analyzed data with help from AT and AM for the yeast experiments; CL performed the MS measurement and data analysis; LS and NH synthesized the peptides; NH synthesized the DiFMUP; MK and LS wrote the manuscript; CM, NV, and CL revised the manuscript.

## Supporting information


**Fig. S1.** Mass spectrometry results of the pull‐down with the Tom6 trap‐peptides and yeast cytosolic fractions.
**Fig. S2.** Interaction between Tom6 and individual subunits of PP4 and PP2A.
**Fig. S3.** Purification and analysis of tagged Psy2reg and Tom6 variants.
**Fig. S4.** Purification and analysis of tagged Cdc55^reg^.
**Fig. S5.** HPLC trace and MS spectrum of Tom6^wt^.
**Fig. S6.** HPLC trace and MS spectrum of Tom6^Pfa16^.
**Fig. S7.** HPLC trace and MS spectrum of Tom6^FxxP→AxxA^.
**Fig. S8.** HPLC trace and MS spectrum of Tom6^N‐term^.
**Fig. S9.** HPLC trace and MS spectrum of Tom6^C‐term^.
**Fig. S10.** HPLC trace and MS spectrum of Control peptide.
**Fig. S11.** HPLC trace and MS spectrum of Tom6^pS16^ (31 aa).
**Fig. S12.** HPLC trace and MS spectrum of Tom6^pS16^ (15 aa).
**Fig. S13.** HPLC trace and MS spectrum of Tom6^pS44^.
**Fig. S14.** HPLC trace and MS spectrum of Bbc1^pS621^.
**Fig. S15.** HPLC trace and MS spectrum of Tom6^PfaNT^.
**Fig. S16.** ^1^H NMR spectrum of DiFMU (1).
**Fig. S17.** NMR spectrum of DiFMUP (2): ^1^H NMR (400 MHz, DMSO, δ): 7.65 (dd, *J* = 2.0, 10.7 Hz, ^1^H), 6.51 (s, ^1^H), 2.41 (d, *J* = 1.2 Hz, 3H).
**Fig. S18.** NMR spectrum of DiFMUP (2): ^31^P NMR (160 MHz, CDCl_3_, δ): −5.71 (s).
**Fig. S19.** MS spectrum of DiFMUP (2): 19F NMR (375 MHz, CDCl3, δ): −130.4, −145.6.
**Fig. S20.** MS spectrum of DiFMUP (2) C10H7F2O6P. Measured m/z by HPLCMS 293.0 [M+H]+ (calculated m/z 293.0 [M+H]+).

**Table S1.** Proteomics data of pulldown with Tom6 trap‐peptides.

**Table S2.** Significantly enriched proteins with Tom6^wt^ vs. Cys.
**Table S3.** Significantly enriched proteins with Tom6^Pfa^ vs. Cys.
**Table S4.** Significantly enriched proteins Tom6^Pfa^ vs. Tom6^wt^.
**Table S5.** Proteomics data of PP4 and PP2A purified from yeast.

## Data Availability

The mass spectrometric raw files as well as the MaxQuant output files have been deposited to the ProteomeXchange Consortium via the PRIDE partner repository and can be accessed using the identifier PXD058384 (https://proteomecentral.proteomexchange.org/cgi/GetDataset?ID=PXD058384).
