# Supplementary material for: Synthetic trap‐peptides identify a TOM complex phosphatase – PP2A dephosphorylates Tom6
Source: FEBS J. 2025 Sep 2;293(1):271–94. doi: 10.1111/febs.70246 (PMC12797000; doi:10.1111/febs.70246)
Supplement: Supplementary file 1 — Fig. S1. Mass spectrometry results of the pull‐down with the Tom6 trap‐peptides and yeast cytosolic fractions. Fig. S2. Interaction between Tom6 and individual subunits of PP4 and PP2A. Fig. S3. Purification and analysis of tagged Psy2reg and Tom6 variants. Fig. S4. Purification and analysis of tagged Cdc55reg. Fig. S5. HPLC trace and MS spectrum of Tom6wt. Fig. S6. HPLC trace and MS spectrum of Tom6Pfa16. Fig. S7. HPLC trace and MS spectrum of Tom6FxxP→AxxA. Fig. S8. HPLC trace and MS spectrum of Tom6N‐term. Fig. S9. HPLC trace and MS spectrum of Tom6C‐term. Fig. S10. HPLC trace and MS spectrum of Control peptide. Fig. S11. HPLC trace and MS spectrum of Tom6pS16 (31 aa). Fig. S12. HPLC trace and MS spectrum of Tom6pS16 (15 aa). Fig. S13. HPLC trace and MS spectrum of Tom6pS44. Fig. S14. HPLC trace and MS spectrum of Bbc1pS621. Fig. S15. HPLC trace and MS spectrum of Tom6PfaNT. Fig. S16. 1H NMR spectrum of DiFMU (1). Fig. S17. NMR spectrum of DiFMUP (2): 1H NMR (400 MHz, DMSO, δ): 7.65 (dd, J = 2.0, 10.7 Hz, 1H), 6.51 (s, 1H), 2.41 (d, J = 1.2 Hz, 3H). Fig. S18. NMR spectrum of DiFMUP (2): 31P NMR (160 MHz, CDCl3, δ): −5.71 (s). Fig. S19. MS spectrum of DiFMUP (2): 19F NMR (375 MHz, CDCl3, δ): −130.4, −145.6. Fig. S20. MS spectrum of DiFMUP (2) C10H7F2O6P. Measured m/z by HPLCMS 293.0 [M+H]+ (calculated m/z 293.0 [M+H]+). [file FEBS-293-271-s001.pdf]

Supporting Information:
Synthetic trap-peptides identify a TOM complex phosphatase – PP2A dephosphorylates Tom6

Laura Scheinost [a,b,c], Christina Ludwig [d,e], Nico Höfflin [a,b], Asli Aras Taskin [f], Adinarayana Marada [f], F.-Nora Vögtle [g,h,i], Chris Meisinger [b,f,*], Maja Köhn [a,b,j,*]

a: Faculty of Biology, Institute of Biology III, University of Freiburg, Freiburg, Germany

b: Signalling Research Centres BIOSS and CIBSS, University of Freiburg, Freiburg, Germany

c: Spemann Graduate School of Biology and Medicine (SGBM), University of Freiburg

d: Chair of Proteomics and Bioanalytics, TUM School of Life Sciences, Technical University of Munich (TUM), Freising, Germany

e: Bavarian Center for Biomolecular Mass Spectrometry (BayBioMS), TUM School of Life Sciences, Technical University of Munich (TUM), Freising, Germany

f: Institute of Biochemistry and Molecular Biology, ZBMZ, Faculty of Medicine, University of Freiburg, Freiburg, Germany

g: Center for Molecular Biology of Heidelberg (ZMBH), DKFZ-ZMBH Alliance, University of Heidelberg, Germany

h: Network Aging Research, Heidelberg University, 69120 Heidelberg, Germany

i: CIBSS - Centre for Integrative Biological Signalling Studies, University of Freiburg,

j: Department of Molecular Cell Biology, Institute for Cell Biology, University of Bonn, Bonn, Germany

* Corresponding authors: chris.meisinger@biochemie.uni-freiburg.de; mkoehn@uni-bonn.de

Table of contents

Supporting figure 1: Related to figure 1

Supporting table 1: Proteomics data of pulldown with Tom6 trap-peptides (separate xls file)

Supporting table 2: Significantly enriched proteins with $Tom6^{wt}$ vs. Cys

Supporting table 3: Significantly enriched proteins with $Tom6^{Pfa}$ vs. Cys

Supporting table 4: Significantly enriched proteins $Tom6^{Pfa}$ vs. $Tom6^{wt}$

Supporting figure 2: Related to figure 2

Supporting figure 3: Related to figure 3

Supporting figure 4: Related to figure 4

Supporting table 5: Proteomics data of PP4 and PP2A purified from yeast (separate xls file)

Supporting figures 5-15: HPLC traces and MS spectra of synthesized peptides

Supporting figures 16-20: NMR and MS spectra of the synthesized DiFMUP

A

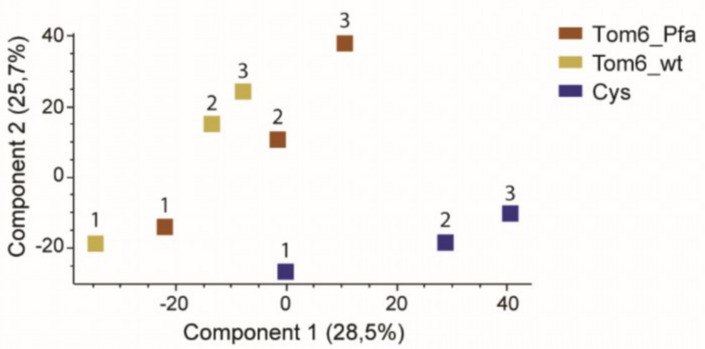

B

Sequence of identified Tom6 peptides	Intensity Cys_1	Intensity Cys_2	Intensity Cys_3	Intensity Tom6_wt_ 1	Intensity Tom6_wt_ 2	Intensity Tom6_wt_ 3	Intensity Tom6_Pfa 1	Intensity Tom6_Pfa 2	Intensity Tom6_Pfa 3
DGMFAMPGAAAGAASPQQPK	0	0	0	3,15E+10	2,47E+10	1,43E+10	0	0	0
DGMFAMPGAAAGAASPQQPKSR	0	0	0	0	0	0	0	0	0
MDGMFAMPGAAAGAASPQQPK	0	0	0	2,56E+10	2,46E+10	1,67E+10	0	0	0
MDGMFAMPGAAAGAASPQQPKSR	0	0	0	4,69E+08	1,99E+08	4,45E+08	0	0	0
MDGMFAMPGAAAGAASPQQPKSRFQAFK	0	0	0	0	0	1,12E+08	0	0	0
SRFQAFK	0	0	0	4,75E+10	4,42E+10	9,36E+09	4,92E+10	3,73E+10	2,40E+10

Lowest value — Highest value

C

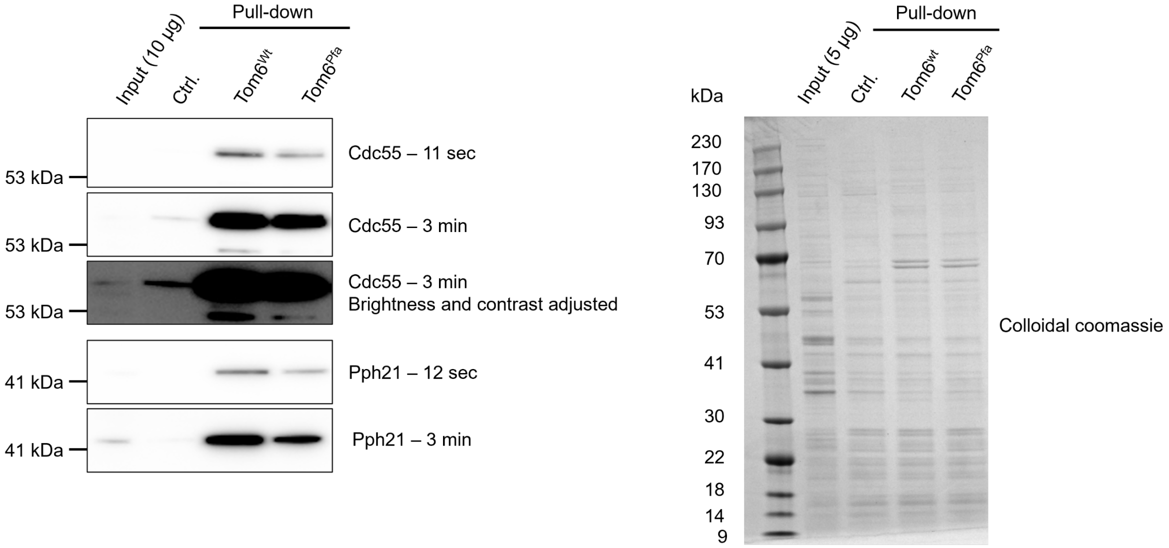

Supporting figure 1: Mass spectrometry results of the pull-down with the Tom6 trap-peptides and yeast cytosolic fractions. The pull-downs were performed in triplicates with the Tom6^{wt} and Tom6^{Pfa} trap peptides. As a negative control cysteine was coupled to the beads instead of the peptides. **A** Principal component analysis of the mass spectrometry results. The numbers 1-3 represent the replicates. **B** Intensities of the different Tom6 peptides found by mass spectrometry in each replicate. The mass of Pfa was not defined in the database search, thus only peptides containing the wild-type serine could be detected. **C** Western blots shown in Fig. 1G including the input and different exposure times. SDS-gel of the pull-down stained with colloidal coomassie. Ctrl.: control.

Supporting table 1: (separate xls file) Mass spectrometry results of the pull-down with trap-peptides and with the control beads from yeast cytosolic fractions.

Supporting table 2: Significantly enriched proteins with Tom6^{wt} compared to the cysteine negative control. The found proteins are sorted from highest to lowest $\log_2$ fold change.

Gene Name	$-\log_{10}$ p-value	$\log_2$ fold change
SSB1	3.3575	13.7703
TOM6	3.0925	10.0194
NUP120	2.3377	5.7050
PSY2	3.4773	5.6213
LOS1	2.7698	5.4847
SEC24	1.9414	5.3605
MTR10	1.9775	5.2203
KAP120	1.5861	5.1141
KAP122	1.8915	5.0407
MSN5	3.0389	4.8258
WHI2	2.7905	4.5679
NTA1	2.3266	4.526
PPH3	2.5879	4.4029
NUP84	3.2227	4.3885
WTM2	4.6897	4.0634
SSK2	2.5149	3.8761
ISN1	2.4655	3.8238
PDI1	2.4963	3.6078
YBP1	2.2547	3.5572
UBP15	3.7971	3.4656
YFR006W	3.4227	3.3979
CDC55	3.1243	3.3437
NMD5	2.3854	3.1463
ARP6	2.5717	3.0720
TAE1	1.9382	3.0701
KAP104	2.8180	2.9851
FUS3	2.2639	2.9492
CWH41	3.0742	2.9090
PSY4	1.9403	2.8696
MDM20	2.6392	2.6783
TUB4	2.6882	2.6630
SXM1	2.4278	2.6122
GEA2	2.0169	2.5858
HRP1	2.9455	2.5381
TPD3	3.0903	2.5233
BIM1	1.5708	2.5098
SEC23	2.6683	2.4951
BUD7	2.0730	2.3233
NUP85	1.6984	2.2918
KAP95	2.3427	2.2769

	$-\log_{10}$ p-value	$\log_2$ fold change
OPI10	2.7440	2.2689
SWD2	1.7088	2.2667
CYC8	2.5638	2.2484
ADH6	3.026	2.2036
SAP185	1.8820	2.1888
PPH22	2.9293	2.1821
PCD1	2.3228	2.1691
CSM1	2.8863	2.0253
CPR1	2.7444	1.9755
PPH21	2.4676	1.9640
UBP12	1.7458	1.9201
MET18	1.7872	1.8820
KAP123	2.3544	1.8816
YKE2	2.1937	1.8144
RPO31	2.0425	1.7381
PSR1	2.8127	1.6631
RTS1	2.0192	1.6293
KAP114	2.2741	1.6100
KAR2	1.8512	1.5894
COP1	1.8362	1.5772
GRE2	2.6127	1.5644
PSE1	3.0142	1.5393
SRP1	1.7174	1.5392
UBR1	2.0583	1.4658
YGR017W	2.5544	1.4565
DDR48	3.7067	1.4530
POL30	1.9855	1.4425
SAP155	1.7132	1.3856
TFC1	3.3346	1.3749
SEC27	2.3024	1.3702
GLC3	2.2973	1.2516
UFD4	2.6299	1.2464
RUP1	2.8694	1.2431
ELP3	2.0862	1.2263
BUD14	2.0414	1.1978
HOG1	2.2125	1.1745
SSE1	2.4878	1.1673
IKI3	2.2987	1.0933
RPB8	3.9111	1.0645
ZPR1	3.3329	0.9883

Gene Name	$-\log_{10}$ p-value	$\log_2$ fold change
NTF2	1.8973	0.9781
RPB4	2.2395	0.9670
SNF1	2.3966	0.9443
RPG1	2.3336	0.9289
ELP2	2.7565	0.8810
SQT1	2.0096	0.8623
CPR5	2.0396	0.8277
RPB7	2.3396	0.7674
TIF5	2.4795	0.7148
OCA1	2.9166	0.6881

Supporting table 3: Significantly enriched proteins with Tom6^{Pfa} compared to the cysteine negative control. The found proteins are sorted from highest to lowest log_2 fold change.

Gene Name	$-log_{10}$ p-value	log_2 fold change	Gene Name	$-log_{10}$ p-value	log_2 fold change
SSB1	7.0954	15.4735	KAP104	3.3544	2.0669
YFR006W	4.5460	5.7128	OPI10	3.7175	2.0372
NTA1	4.3809	5.4768	CSM1	3.1795	1.7921
PSY2	2.9255	5.3439	CPR5	2.8818	1.7052
MSN5	3.2298	4.9175	SSA3	2.8140	1.6956
YBP1	2.0462	4.8510	CRG1	2.8148	1.6663
LOS1	2.5058	4.6014	GRE2	2.7978	1.6660
TAE1	2.5490	4.3142	ATE1	3.7887	1.6092
KAP120	1.6961	4.2952	FRD1	1.9309	1.5833
PPH3	3.0440	4.2705	SXM1	2.0819	1.5571
KAP122	1.4697	4.0461	SSA4	3.9332	1.4784
MTR10	1.5196	3.8848	PTC7	2.3876	1.4469
SEC24	1.7115	3.8691	YGR017W	3.2514	1.3400
YJR008W	1.7821	3.7385	SSE1	3.4584	1.3066
WHI2	3.5759	3.5918	KAP95	1.8011	1.1859
CDC55	3.7960	3.5328	KAP114	1.8091	1.1811
PDI1	3.2657	3.5047	CSE1	1.8308	1.1387
PSY4	3.4037	3.4995	SSA2	1.9435	1.1337
ISN1	2.6237	3.1775	KAP123	1.9309	1.1242
PCD1	2.9724	2.9608	PSR1	2.0078	1.0444
FUS3	3.5976	2.8909	HOG1	2.1303	1.0227
MDM20	1.7114	2.8342	OYE3	2.2082	1.0138
ESS1	1.4872	2.7813	GPN3	2.6558	0.9795
NUP120	1.9625	2.7743	RKM4	2.1330	0.9283
UBP15	3.0403	2.7566	HRP1	2.3599	0.8452
SSC1	2.3349	2.6860	CIA1	2.4171	0.7722
ARP6	2.0134	2.6120	CMK2	2.8563	0.6922
SWD2	1.9436	2.4568			
CYC8	3.4551	2.4489			
NMD5	2.0608	2.3835			
ADH6	1.9993	2.3686			
PPH22	4.4603	2.2949			
TPD3	3.4188	2.2922			
KAR2	3.1365	2.2318			
SSB2	2.9619	2.2170			
CPR1	2.9159	2.1615			
ECM15	1.6538	2.1261			
PPH21	3.5259	2.1109			
WTM2	2.7666	2.0954			
SDH1	2.3777	2.0897			

Supporting table 4: Significantly enriched proteins with Tom6^{Pfa} and Tom6^{wt} compared to each other. The found proteins are sorted from highest to lowest log_2 fold change.

Significantly enriched proteins with Tom6^{Pfa}

Gene Name	$-log_{10}$ p-value	log_2 fold change
ESS1	1.7848	3.3819
CYC1	3.2431	2.8888
MCK1	3.1398	2.8027
ATP4	1.6940	2.3573
GTT1	1.6306	2.3429
YFR006W	2.7365	2.3148
ALD2	3.1363	2.2923
ALD3	1.7485	2.0353
NSP1	2.4702	2.0341
CFD1	2.1186	1.9120
YPR084W	1.8639	1.8564
TAE1	2.6450	1.4521
GCS1	1.9632	1.4472
APD1	2.0128	1.2952
YPT7	3.3277	0.9058
ARL1	2.3003	0.8161
ARP2	3.9281	0.7890
GYP7	3.3792	0.6854
RPC40	3.9832	0.5572
TSA2	4.4572	0.5006

Significantly enriched proteins with Tom6^{wt}

Gene Name	$-log_{10}$ p-value	log_2 fold change
TOM6	3.5578	-9.7685
SSK2	2.7492	-3.9545
NUP84	2.3598	-2.9635
HPA2	2.3674	-2.7161
RPO31	4.2614	-2.3398
RIM15	3.1026	-1.9251
HRP1	2.2053	-1.6928
NUP85	2.4726	-1.4972
MTR10	2.2816	-1.4049
RPB7	1.8599	-1.3187
DIS3	1.9043	-1.1745
SEC24	2.8085	-1.1621
RPB4	2.5534	-1.1605
RPS23B	2.6110	-1.1511
ELP3	1.9911	-1.1461
ARX1	1.9878	-1.1247
WTM2	2.6600	-1.0839
IKI3	2.6621	-1.0719
ELP2	3.0489	-1.0203
CDC73	3.7699	-0.9390
PWP1	2.7104	-0.9346
DDR48	2.6184	-0.8394
OCA1	2.2737	-0.8238
ESC1	2.1779	-0.8197
RUP1	2.1501	-0.8011
PSE1	2.9965	-0.7436
BUD7	2.2429	-0.7280
RPS20	2.3239	-0.6777
TIF5	3.5254	-0.5836
ZPR1	2.8370	-0.5459
KAP114	3.6270	-0.4288

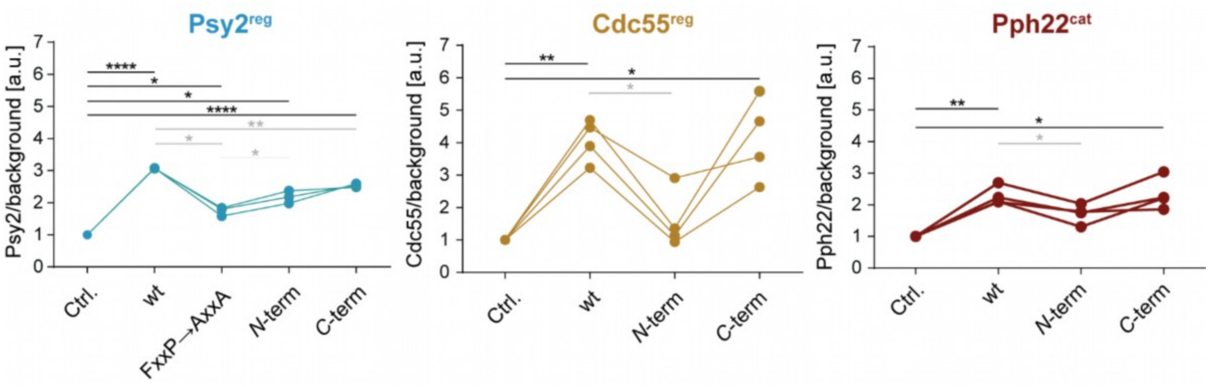

Supporting figure 2: Interaction between Tom6 and individual subunits of PP4 and PP2A. All graphs from Figure 2 are included for which significant differences were found not only to the control (Ctrl.) but also between the different peptides when analyzing the data with a one-way ANOVA followed by a Tukey's multiple comparisons test. All significant differences are indicated. *: p ≤ 0.05, **: p ≤ 0.01, ***: p ≤ 0.001, ****: p ≤ 0.0001.

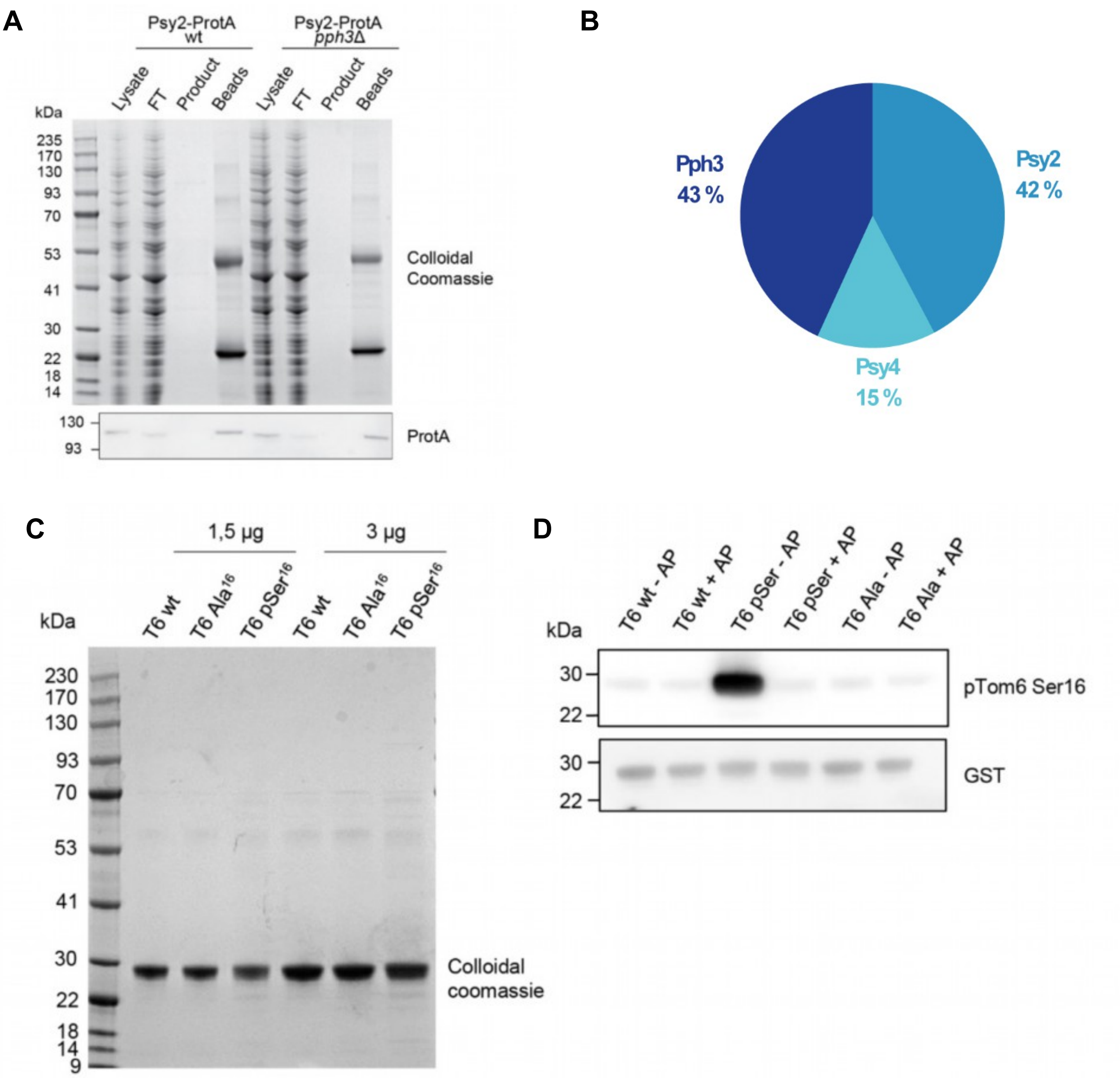

Supporting figure 3: Purification and analysis of tagged Psy2^{reg} and Tom6 variants. **A** Coomassie stained SDS-gel and Western blot against ProtA of the purification steps. ProtA tagged Psy2^{reg} was purified from wild-type (wt) or pph3Δ yeast grown in YPD medium. In the first step, ProtA tagged Psy2^{reg} was bound to IgG beads and the flow-through (FT) collected to confirm binding to the beads. Next, TEV protease was added to cleave the ProtA tag from Psy2^{reg} and release the products Psy2^{reg}/Pph3^{cat} or Psy2^{reg} *pph3Δ*. The TEV protease was removed by addition of Ni-NTA beads and the products collected in the flow-through. The beads were boiled in sample buffer to visualize the uncleaved product. **B** Approximate relative stochiometric amounts of the PP4 subunits in the purified Psy2^{reg}/Pph3^{cat} based on the iBAQ values (Supporting table 5). The mass spectrometry data is not quantitative; thus, these are only approximate ratios between the different subunits. PP4 can either assemble into a dimer containing Pph3^{cat} and Psy2^{reg} or a trimer with Pph3^{cat}, Psy2^{reg} and Psy4^{reg}. **C** SDS-gel stained

with colloidal coomassie of the purified GST-Tom6 proteins. The Tom6 cytosolic domain was expressed with a GST and a His tag in *E.coli*. Three variants were expressed: the wild type (wt) sequence, and the sequence with an alanine or a pSer at position 16. **D** The three GST-Tom6 variants were incubated at 30 °C for 1 h with or without alkaline phosphatase (AP). The phosphorylation levels were detected with pTom6 Ser16 and GST antibodies.

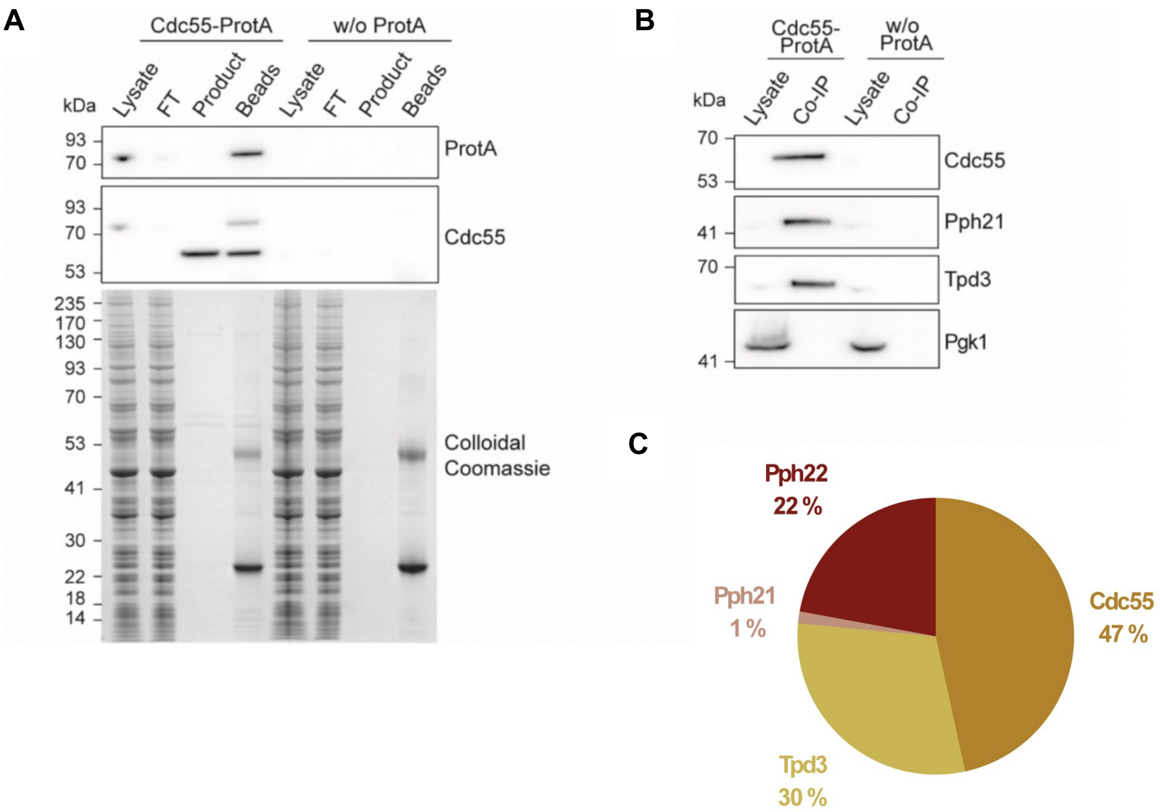

Supporting figure 4: Purification and analysis of tagged Cdc55^{reg}. **A** ProtA tagged Cdc55^{reg} expressing BY4741 yeast was grown in YPD medium and Cdc55^{reg} purified by IgG bead pulldown. As negative control the purification was done in parallel with BY4741 wt yeast without ProtA tag (w/o ProtA). The purified product was cleaved off the beads by TEV protease. Purification was monitored by coomassie stained SDS-gel and Western blot with ProtA and Cdc55 binding antibodies. **B** A Western blot was performed to determine the composition of the purified Cdc55-ProtA (Cdc55^{reg}/Pph22^{cat}) and the control (w/o ProtA) with antibodies against Cdc55, Pph21, Tpd3 and Pgk1. **C** Approximate relative stochiometric amounts of the PP2A subunits in the purified Cdc55^{reg}/Pph22^{cat} based on the iBAQ values (Supporting table 5). The mass spectrometry data is not quantitative; thus, these are only approximate ratios between the different subunits.

Supporting table 5: (separate xls file) Mass spectrometry results of PP2A and PP4 holoenzymes purified from yeast by tagging Cdc55 and Psy2 respectively.

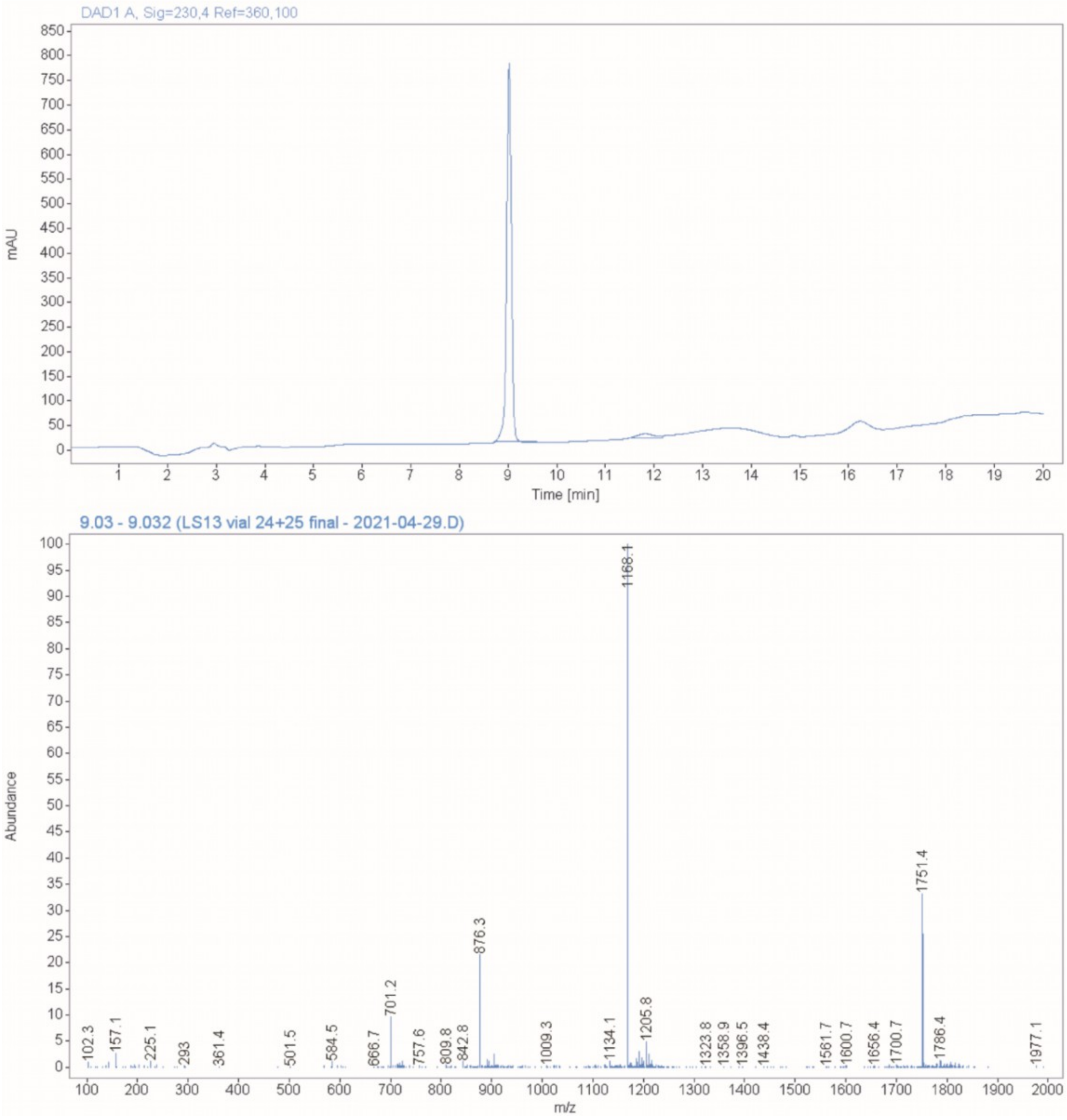

Supporting figure 5: HPLC trace and MS spectrum of Tom6[wt]. HPLC was performed with a gradient of 10 – 90 % acetonitrile in H_2O and UV detection at 230 nm. ESI-MS spectrum with found m/z of 1751.4 $[M+2H]^{2+}$ (calculated m/z 1750.3 $[M+2H]^{2+}$).

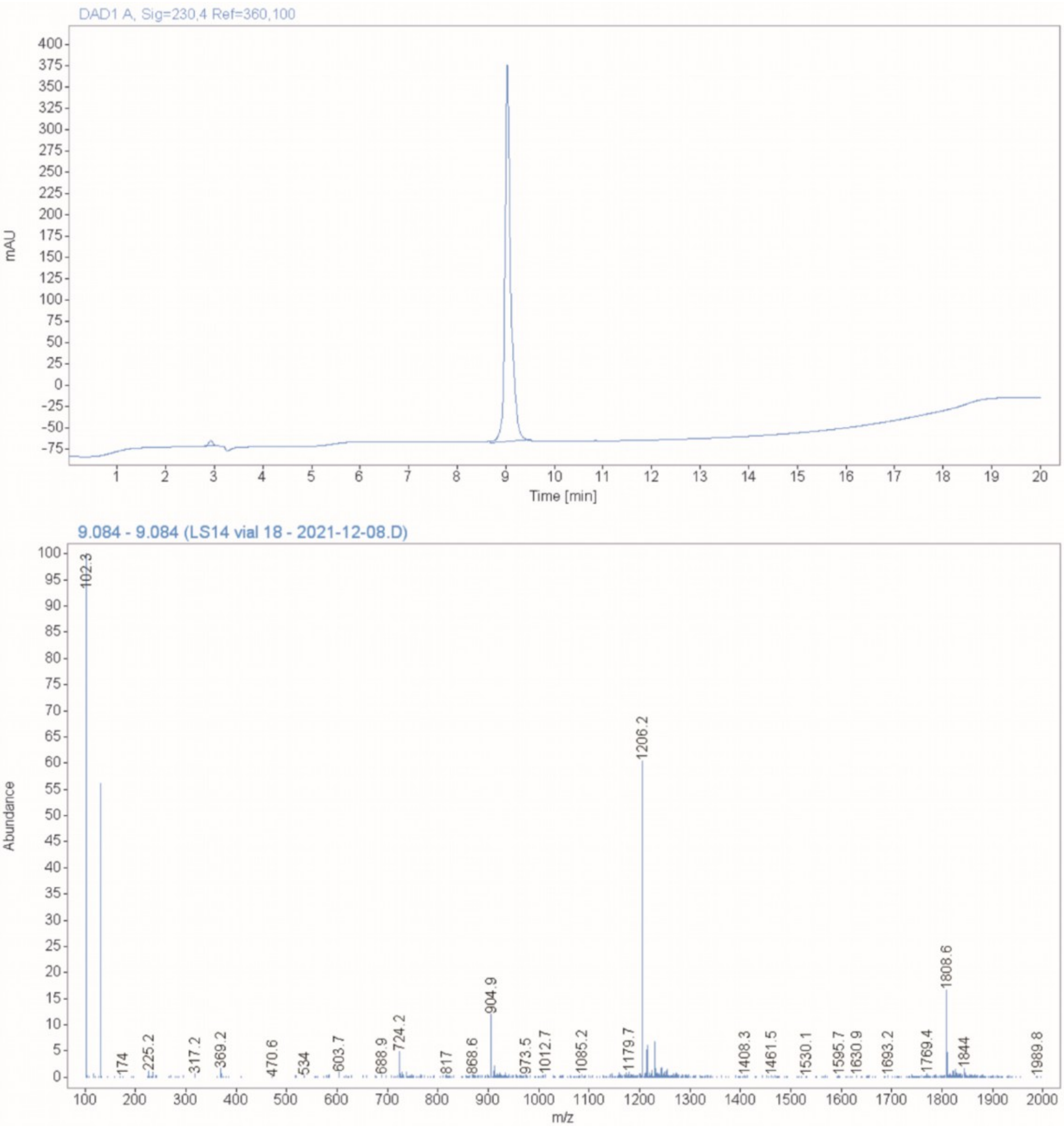

Supporting figure 6: HPLC trace and MS spectrum of Tom6[Pfa16]. HPLC was performed with a gradient of 10 – 90 % acetonitrile in H_2O and UV detection at 230 nm. ESI-MS spectrum with found m/z of 1808.6 $[M+2H]^{2+}$ (calculated m/z 1707.3 $[M+2H]^{2+}$).

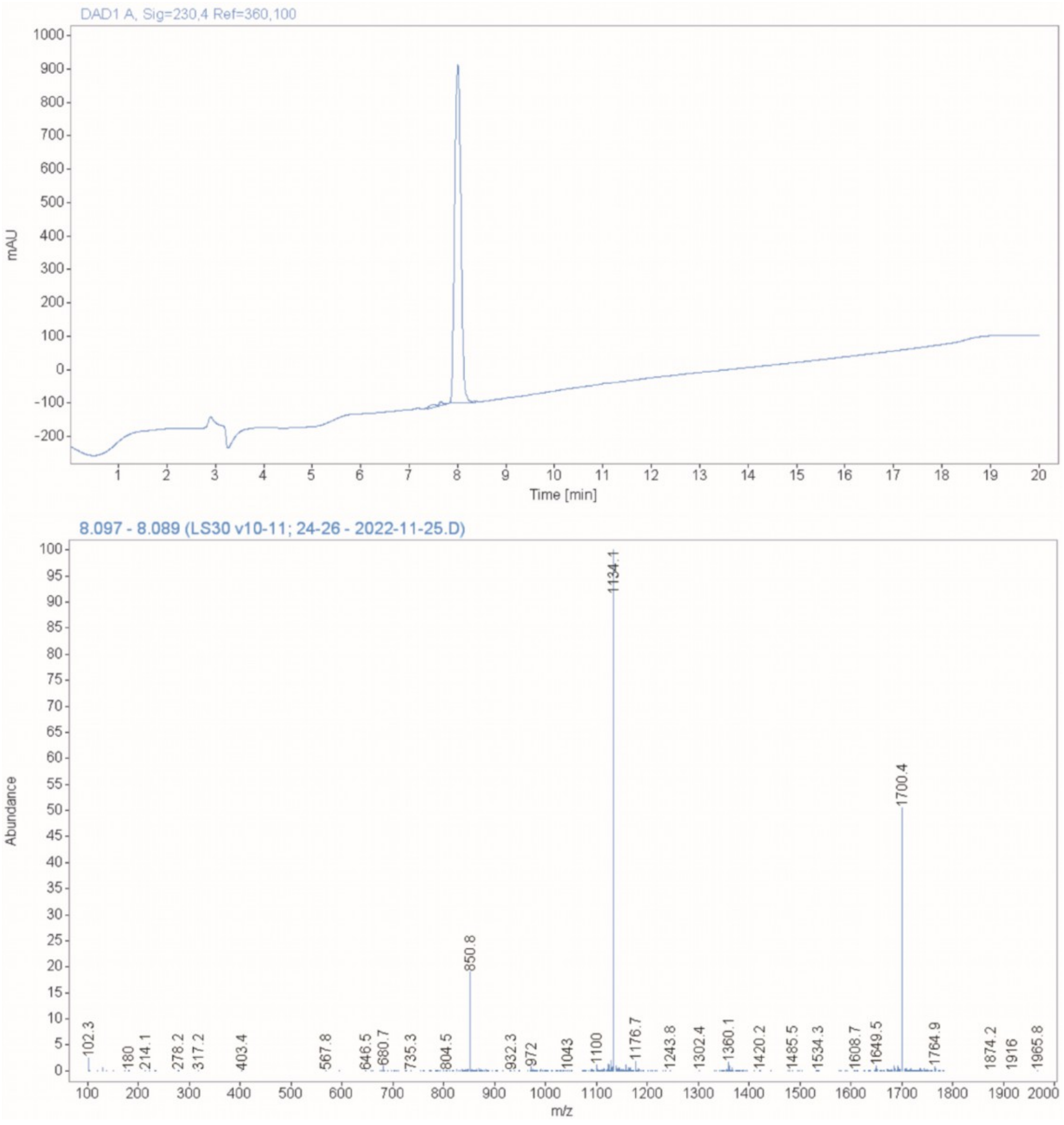

Supporting figure 7: HPLC trace and MS spectrum of Tom6$^{FxxP \rightarrow AxxA}$. HPLC was performed with a gradient of 10 – 90 % acetonitrile in H_2O and UV detection at 230 nm. ESI-MS spectrum with found m/z of 1700.4 $[M+2H]^{2+}$ (calculated m/z 1699.8 $[M+2H]^{2+}$).

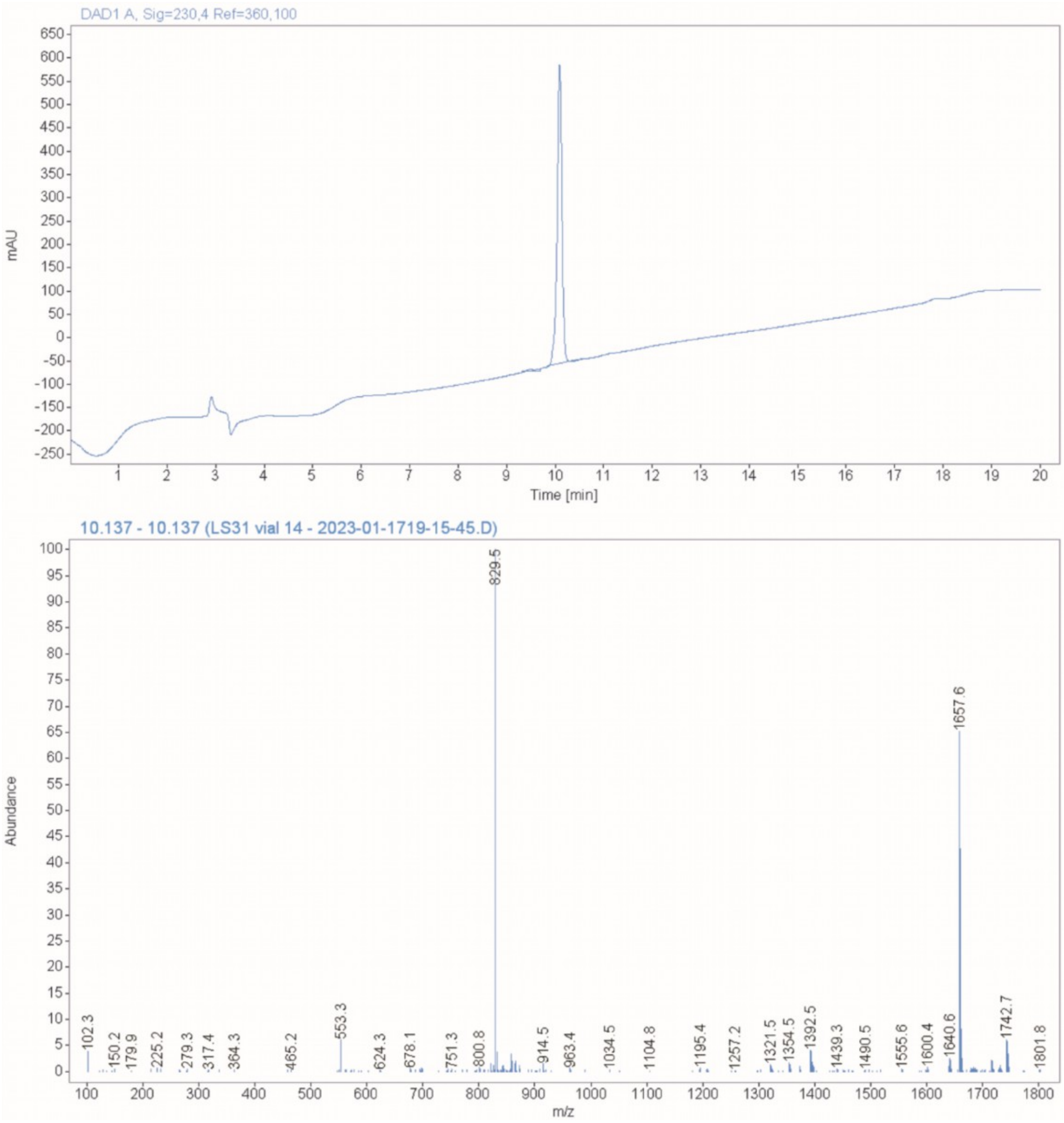

Supporting figure 8: HPLC trace and MS spectrum of Tom6$^{N\text{-term}}$. HPLC was performed with a gradient of 10 – 90 % acetonitrile in H_2O and UV detection at 230 nm. ESI-MS spectrum with found m/z of 1657.6 $[M+H]^+$ (calculated m/z 1656.7 $[M+H]^+$).

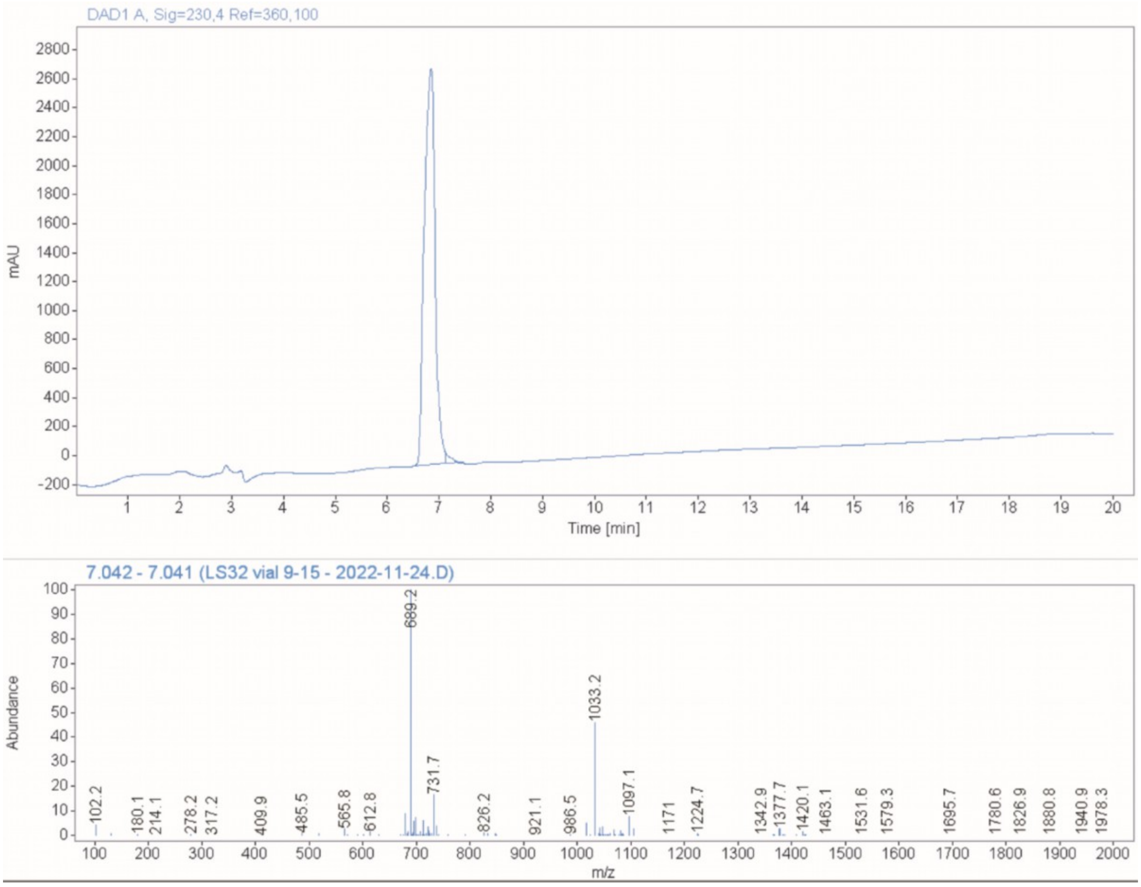

Supporting figure 9: HPLC trace and MS spectrum of Tom6$^{C\text{-term}}$. HPLC was performed with a gradient of 10 – 90 % acetonitrile in H_2O and UV detection at 230 nm. ESI-MS spectrum with found m/z of 1033.2 $[M+H]^+$ (calculated m/z 1032.5 $[M+H]^+$).

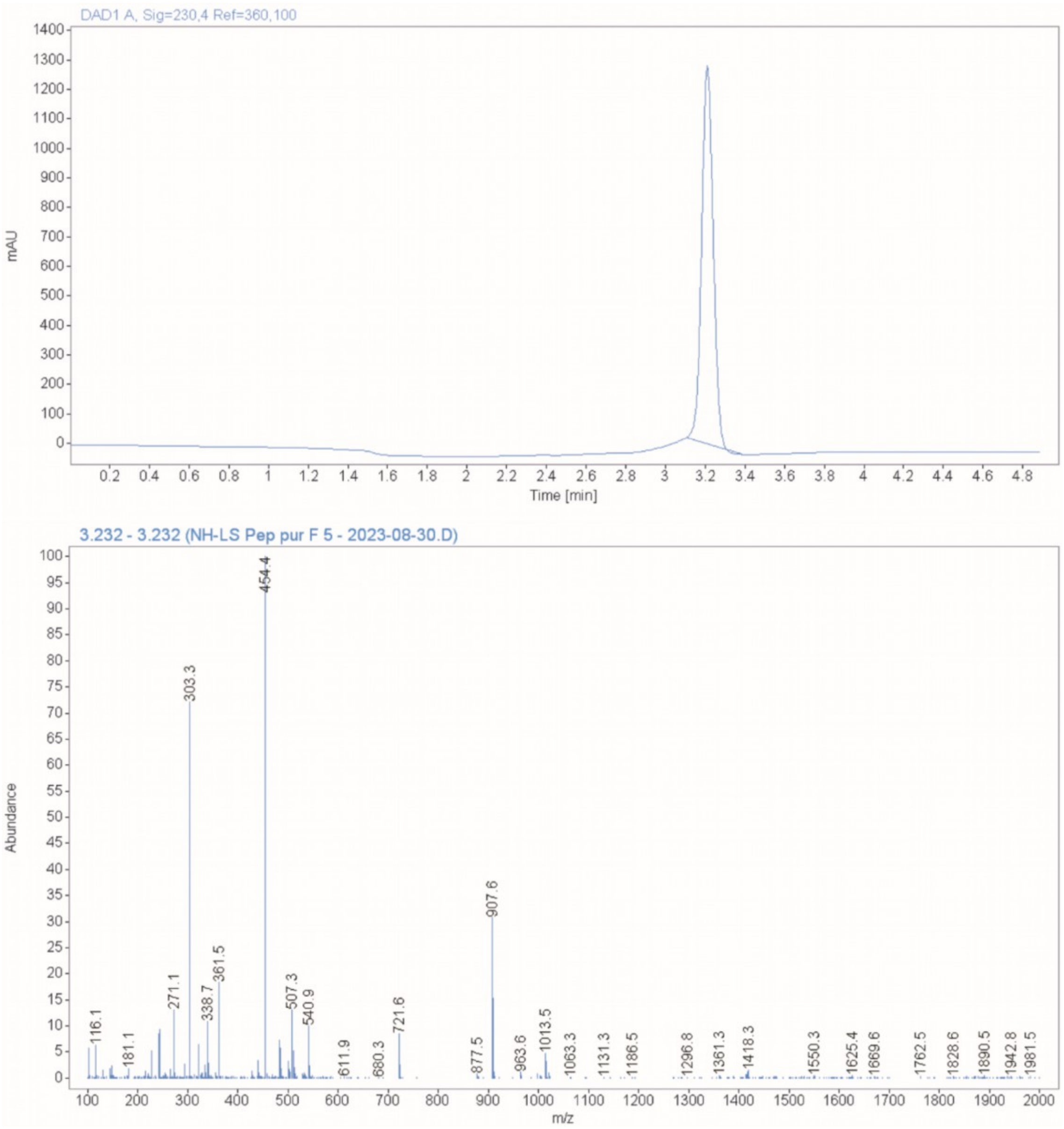

Supporting figure 10: HPLC trace and MS spectrum of Control peptide. HPLC was performed with a gradient of 10 – 90 % acetonitrile in H_2O and UV detection at 230 nm. ESI-MS spectrum with found m/z of 907.6 $[M+H]^+$ (calculated m/z 907.5 $[M+H]^+$).

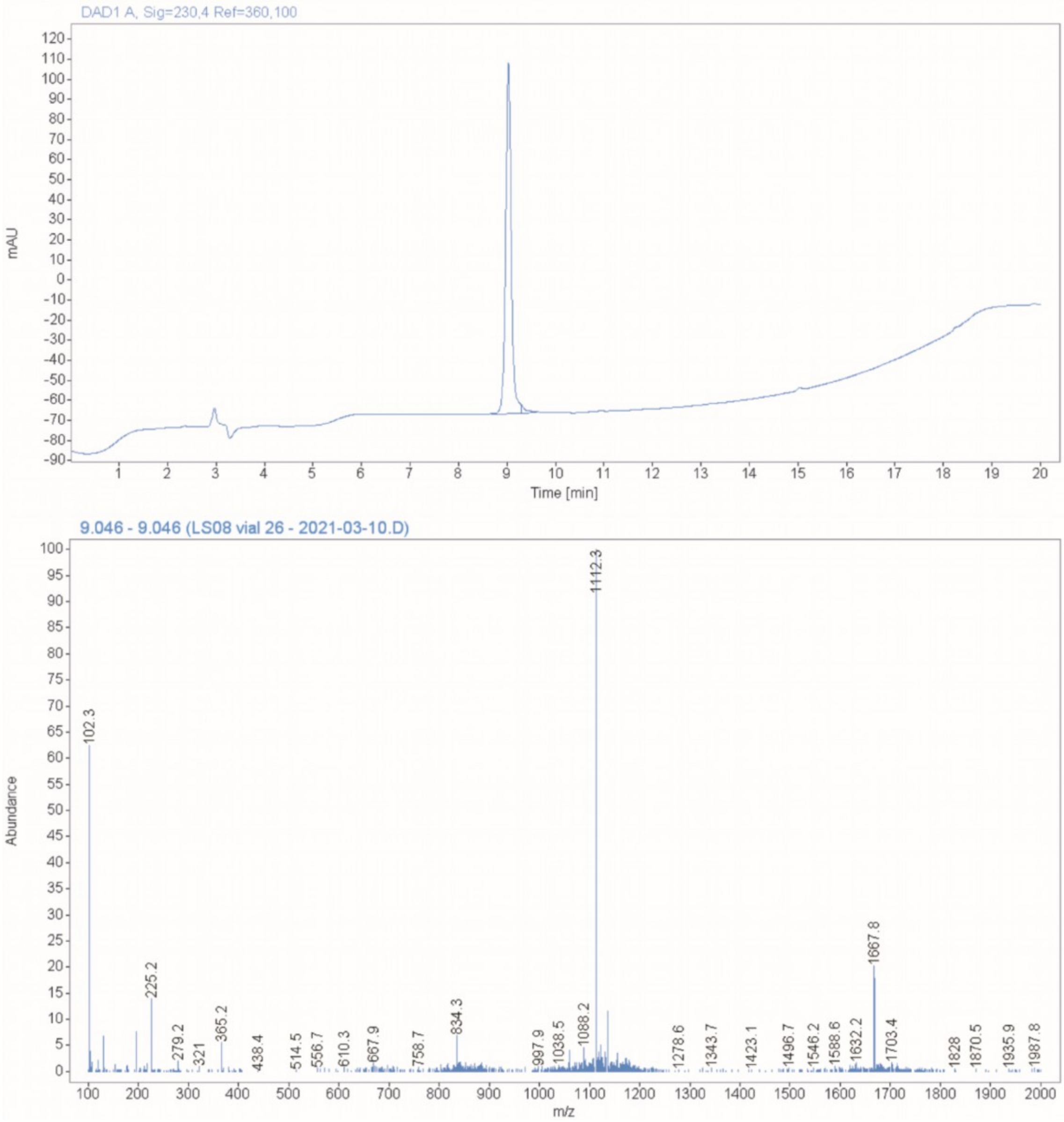

Supporting figure 11: HPLC trace and MS spectrum of Tom6^{pS16} (31 aa). HPLC was performed with a gradient of 10 – 90 % acetonitrile in H_2O and UV detection at 230 nm. ESI-MS spectrum with found m/z of 1667.8 $[M+2H]^{2+}$ (calculated m/z 1666.8 $[M+2H]^{2+}$).

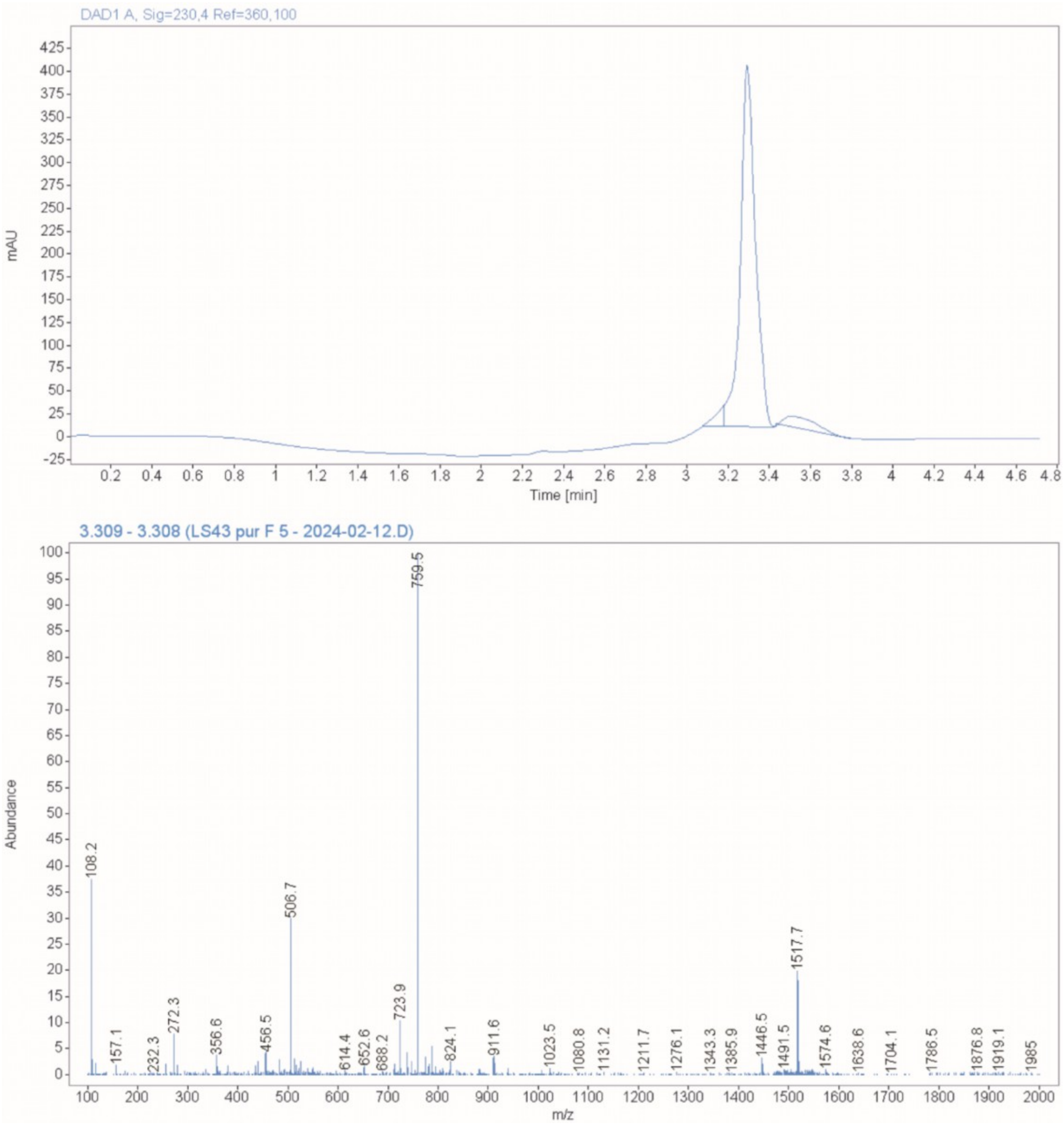

Supporting figure 12: HPLC trace and MS spectrum of Tom6^{pS16} (15 aa). HPLC was performed with a gradient of 10 – 90 % acetonitrile in H_2O and UV detection at 230 nm. ESI-MS spectrum with found m/z of 1517.7 $[M+H]^+$ (calculated m/z 1517.7 $[M+H]^+$).

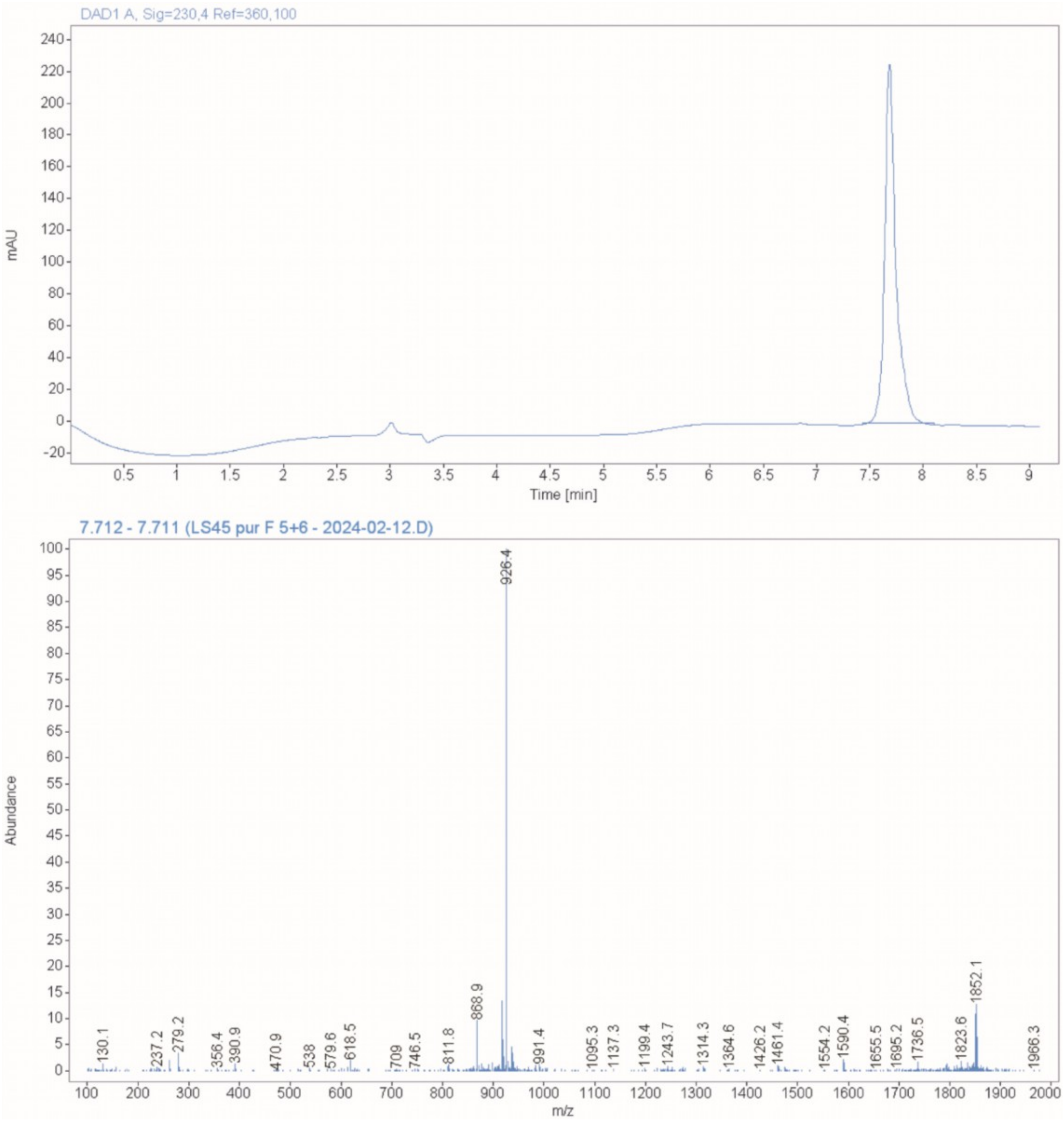

Supporting figure 13: HPLC trace and MS spectrum of Tom22^{pS44}. HPLC was performed with a gradient of 10 – 90 % acetonitrile in H_2O and UV detection at 230 nm. ESI-MS spectrum with found m/z of 1852.1 $[M+H]^+$ (calculated m/z 1851.6 $[M+H]^+$).

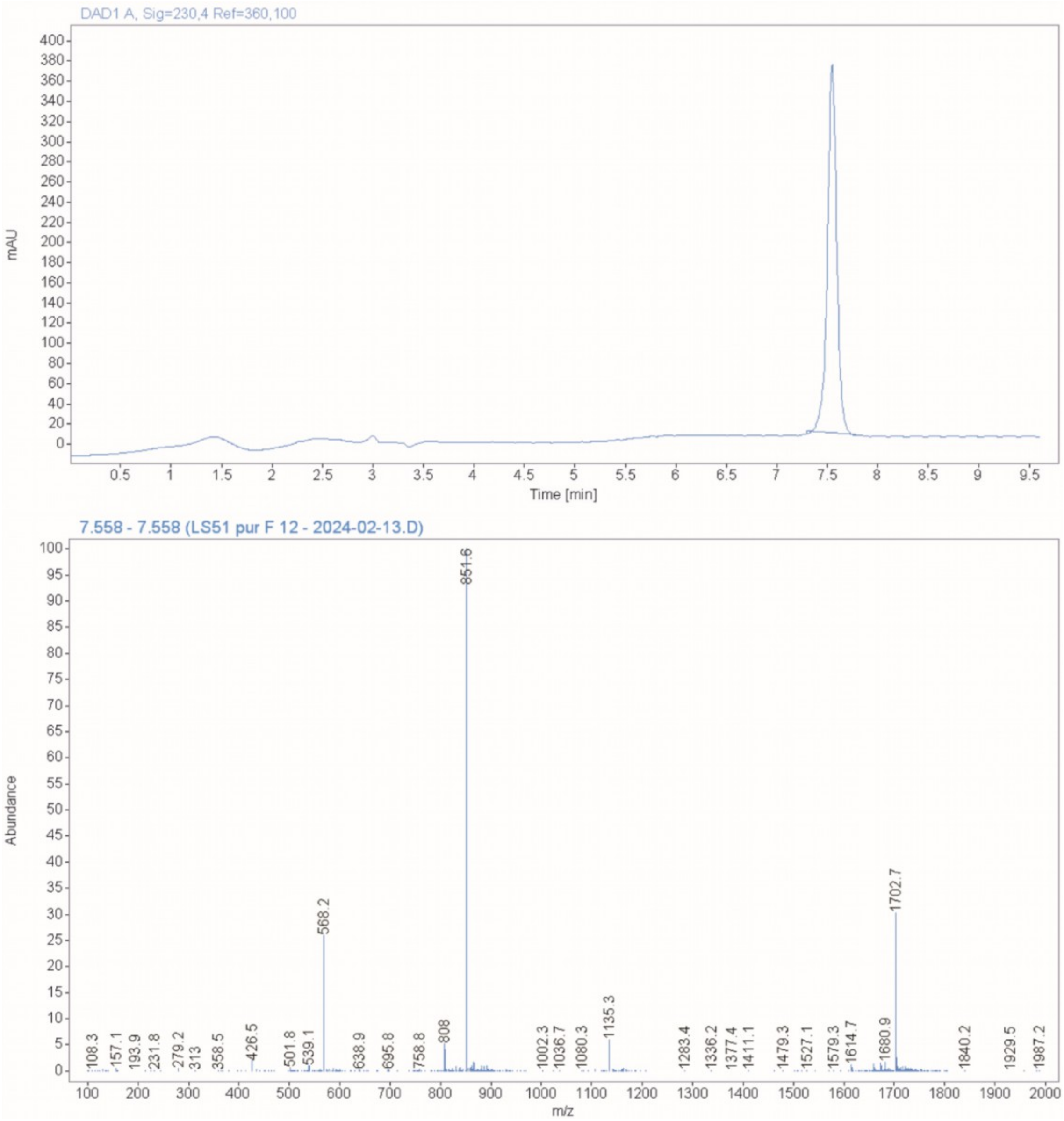

Supporting figure 14: HPLC trace and MS spectrum of Bbc1^{pS621}. HPLC was performed with a gradient of 10 – 90 % acetonitrile in H_2O and UV detection at 230 nm. ESI-MS spectrum with found m/z of 1702.7 $[M+H]^+$ (calculated m/z 1701.9 $[M+H]^+$).

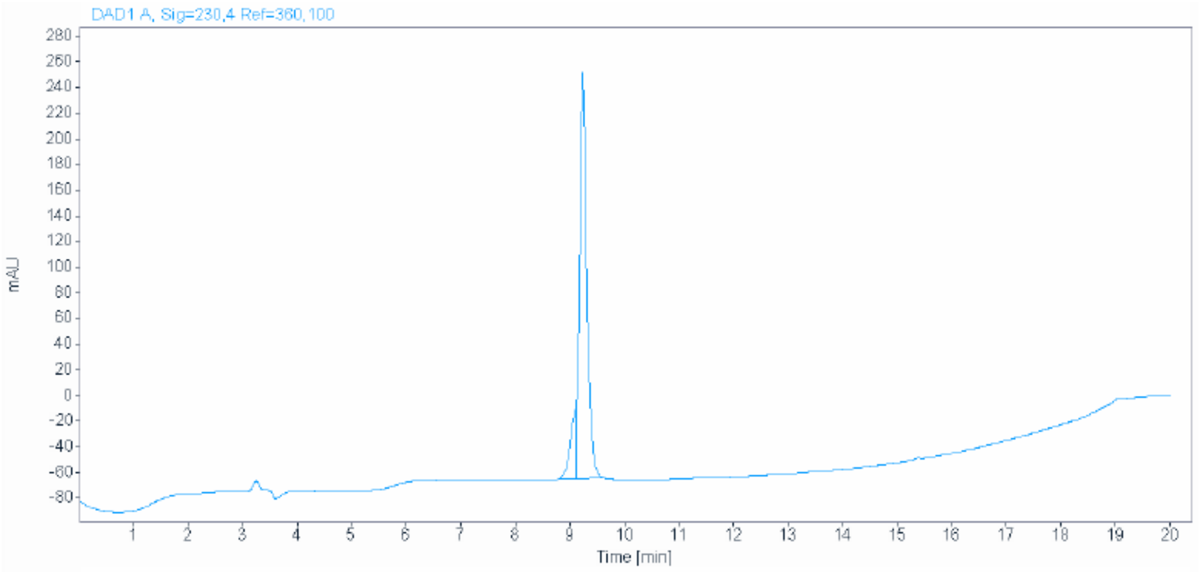

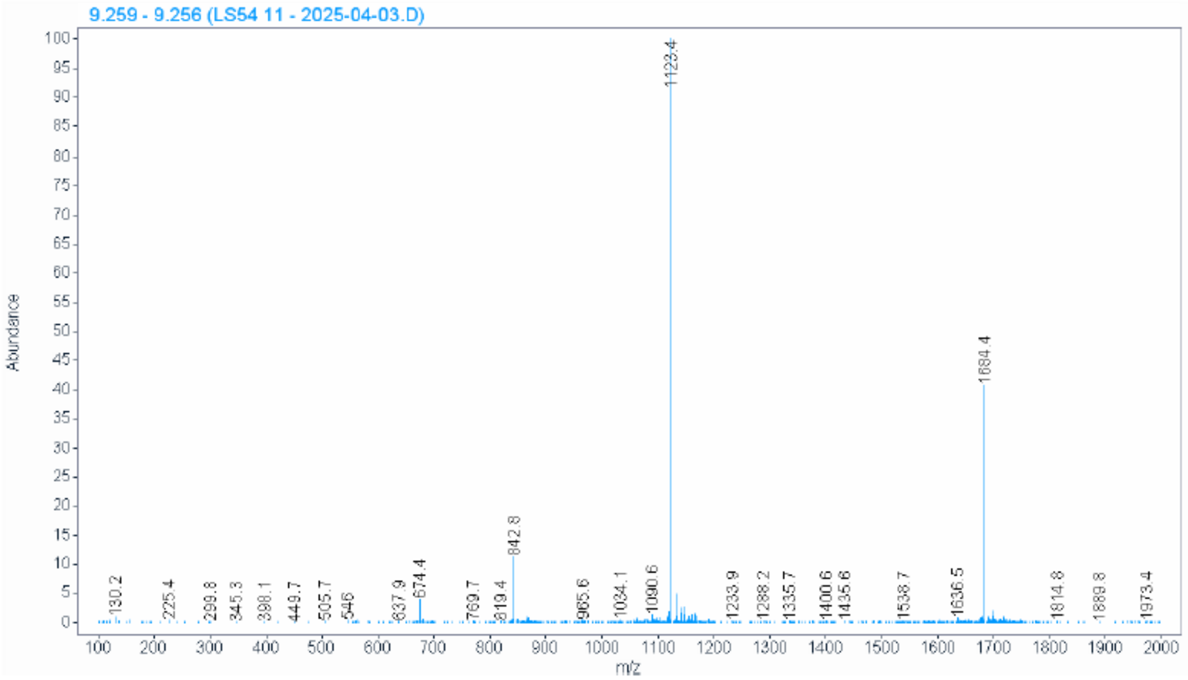

Supporting figure 15: HPLC trace and MS spectrum of Tom6^{PfaNT}. HPLC was performed with a gradient of 10 – 90 % acetonitrile in H_2O and UV detection at 230 nm. ESI-MS spectrum with found m/z of 1684.4[M+2H]$^{2+}$ (calculated m/z 1683.8 [M+2H]$^{2+}$).

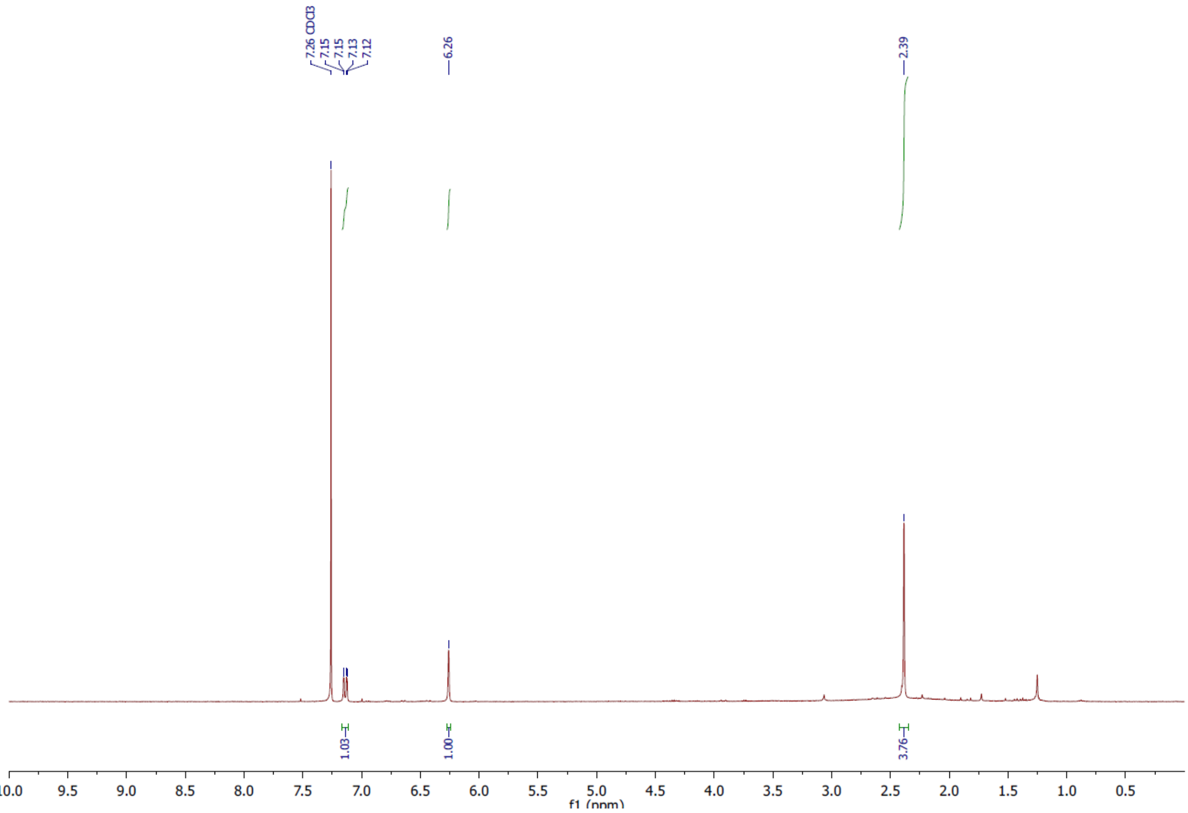

Supporting figure 16: ^{1}H NMR spectrum of DiFMU (**1**). ^{1}**H NMR** (400 MHz, $CDCl_3$, δ): 7.14 (dd, *J* = 2.1, 10.30 Hz, 1H), 6.26 (s, 1H), 2.39 (s, 3H).

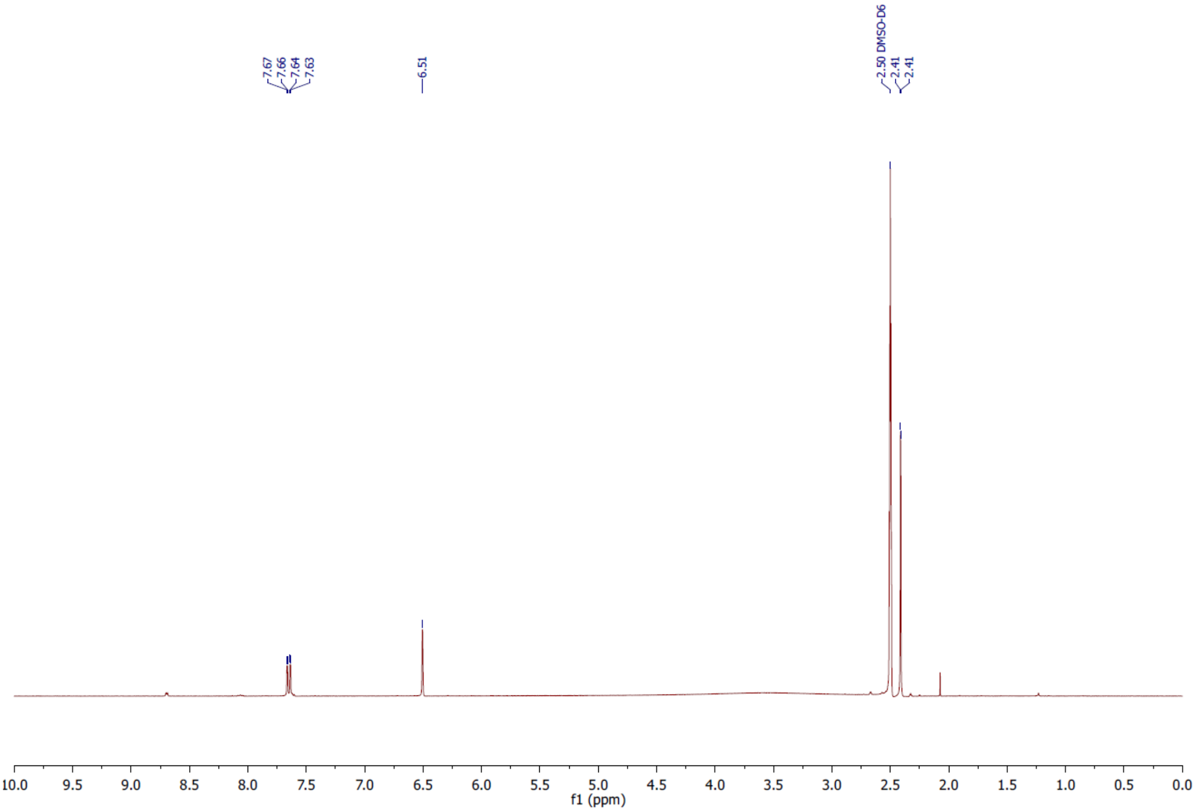

Supporting figure 17: NMR spectrum of DiFMUP (2): ^{1}**H NMR** (400 MHz, DMSO, δ): 7.65 (dd, *J* = 2.0, 10.7 Hz, 1H), 6.51 (s, 1H), 2.41 (d, *J* = 1.2 Hz, 3H).

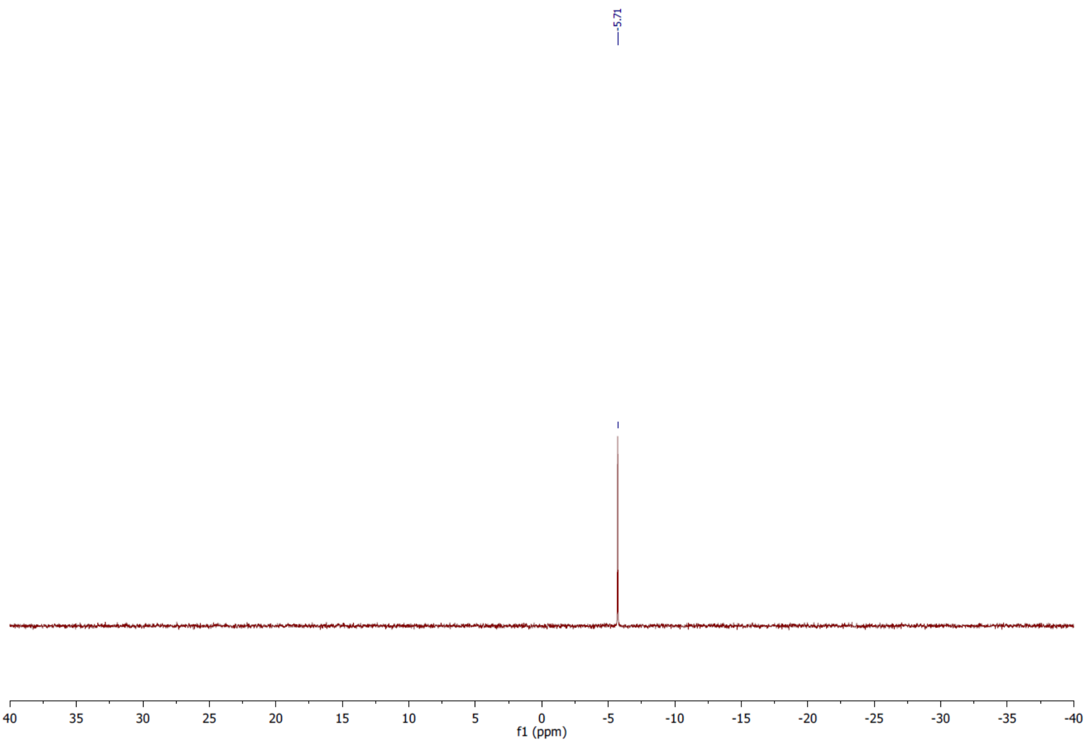

Supporting figure 18: NMR spectrum of DiFMUP (2): **^{31}P NMR** (160 MHz, CDCl3, δ): -5.71 (s).

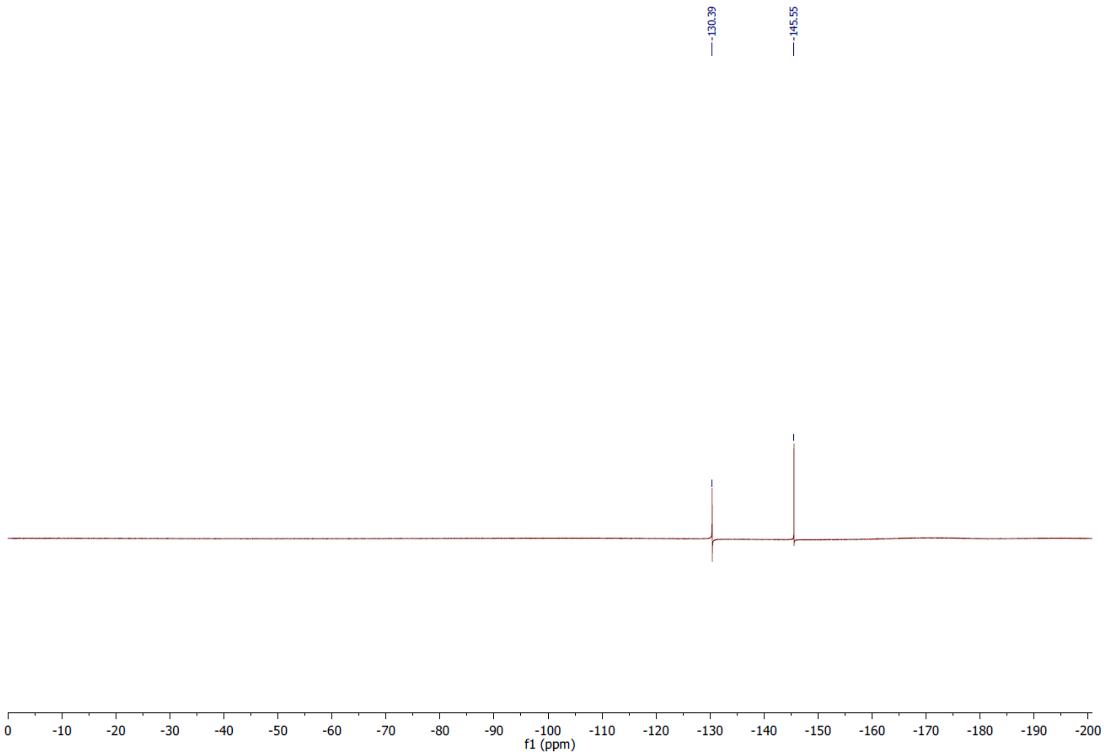

Supporting figure 19: NMR spectrum of DiFMUP (2): **^{19}F NMR** (375 MHz, CDCl3, δ): -130.4, -145.6.

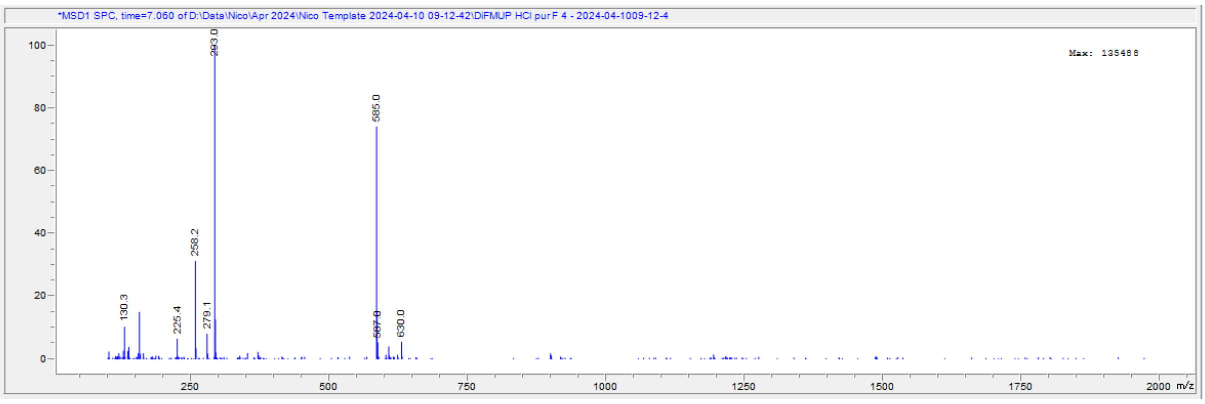

Supporting figure 20: MS spectrum of DiFMUP (2) $C_{10}H_7F_2O_6P$. Measured m/z by HPLC-MS 293.0 $[M+H]^+$ (calculated m/z 293.0 $[M+H]^+$).
